# BODIPY-based fluorescent liposomes with sesquiterpene lactone trilobolide

**DOI:** 10.3762/bjoc.13.128

**Published:** 2017-07-04

**Authors:** Ludmila Škorpilová, Silvie Rimpelová, Michal Jurášek, Miloš Buděšínský, Jana Lokajová, Roman Effenberg, Petr Slepička, Tomáš Ruml, Eva Kmoníčková, Pavel B Drašar, Zdeněk Wimmer

**Affiliations:** 1 Department of Chemistry of Natural Compounds, University of Chemistry and Technology Prague, Technická 5, 166 28 Prague 6, Czech Republic; 2 Institute of Experimental Botany, ASCR, Vídeňská 1083, 142 20 Prague 4, Czech Republic; 3 Department of Biochemistry and Microbiology, University of Chemistry and Technology Prague, Technická 5, 166 28 Prague 6, Czech Republic; 4 Institute of Organic Chemistry and Biochemistry, ASCR, Flemingovo n. 2, 166 10 Prague 6, Czech Republic; 5 Department of Solid State Engineering, University of Chemistry and Technology Prague, Technická 5, 166 28 Prague 6, Czech Republic; 6 Institute of Experimental Medicine, ASCR, Vídeňská 1083, 142 20 Prague 4, Czech Republic; 7 Charles University, Faculty of Medicine in Pilsen, Alej Svobody 76, 323 00 Pilsen, Czech Republic

**Keywords:** BODIPY conjugates, cancer targeting, drug delivery, liposomes, natural compounds, sesquiterpene lactone trilobolide

## Abstract

Like thapsigargin, which is undergoing clinical trials, trilobolide is a natural product with promising anticancer and anti-inflammatory properties. Similar to thapsigargin, it has limited aqueous solubility that strongly reduces its potential medicinal applications. The targeted delivery of hydrophobic drugs can be achieved using liposome-based carriers. Therefore, we designed a traceable liposomal drug delivery system for trilobolide. The fluorescent green-emitting dye BODIPY, cholesterol and trilobolide were used to create construct **6**. The liposomes were composed of dipalmitoyl-3-trimethylammoniumpropane and phosphatidylethanolamine. The whole system was characterized by atomic force microscopy, the average size of the liposomes was 150 nm in width and 30 nm in height. We evaluated the biological activity of construct **6** and its liposomal formulation, both of which showed immunomodulatory properties in primary rat macrophages. The uptake and intracellular distribution of construct **6** and its liposomal formulation was monitored by means of live-cell fluorescence microscopy in two cancer cell lines. The encapsulation of construct **6** into the liposomes improved the drug distribution in cancer cells and was followed by cell death. This new liposomal trilobolide derivative not only retains the biological properties of pure trilobolide, but also enhances the bioavailability, and thus has potential for the use in theranostic applications.

## Introduction

Targeted (smart) drug delivery is a method for specific delivering of an active compound preferentially to some cells or tissues in the human body. This approach has become the key issue for surpassing the bottleneck of drug discovery. With the advent of new technologies and deeper understanding of the biological processes, the concept of specific targeting has become one of the most attractive directions in the field of biomedicine. Specific drug targeting can be achieved by using, for example, antibodies, peptides, polyethylene glycol polymers, and last but not least, liposomes, which have been nowadays extensively investigated [1–2]. In general, liposomes are employed in order to enhance the therapeutic index of an applied drug by improvement of drug absorption, prolonging its biological half-life or decreasing its metabolism [3].

Since “seeing is believing”, it is strongly desired to not only target a drug to the disease-affected tissue, but also image its localization and possibly its mechanism of action directly on the given site. Based on this approach, multimodal agents delivered using a vehicle containing a drug capable of both imaging and curing were developed [4]. A meaningful information about biomolecule/drug localization and action can be gained employing fluorescence imaging, since it provides non-invasiveness, sensitivity and good spatio-temporal resolution altogether [5]. From the plethora of known fluorescent compounds, there are widely used small organic fluorophores, such as BODIPY dyes.

BODIPYs are fluorescent dyes based on the 4,4-difluoro-4-bora-3a,4a-diaza-*s*-indacene scaffold, which have recently experienced increased attention in chemistry [6–10] and life science applications [11–13]. On the grounds of high fluorescence quantum yield, narrow spectral characteristics, and sufficient chemical stability, BODIPYs have been utilized for example as laser dyes, tags of small organic molecules [14–16], drugs [17], cell organelle markers, for antibody, peptide and nucleic acid labelling [18–20], for pH [21], metal [22–23] and redox potential sensing (well-reviewed in Boens et al. [24]), as well as for the development of photodynamically active agents [25–26].

**Figure 1:**
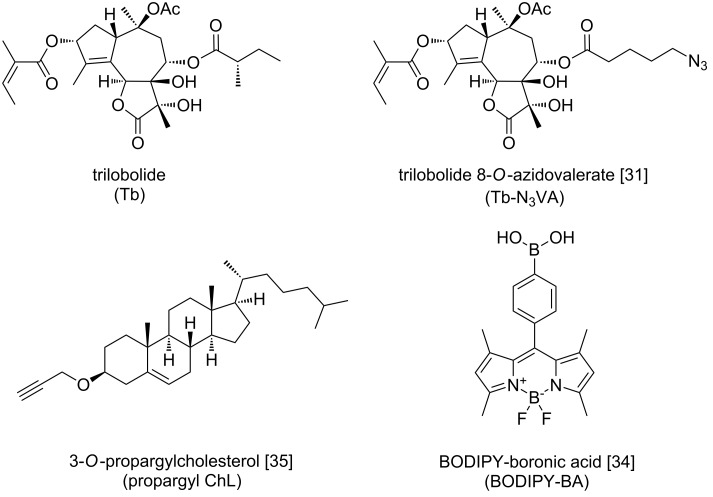
Chemical structures of the basic compounds used in this study.

**Scheme 1:**
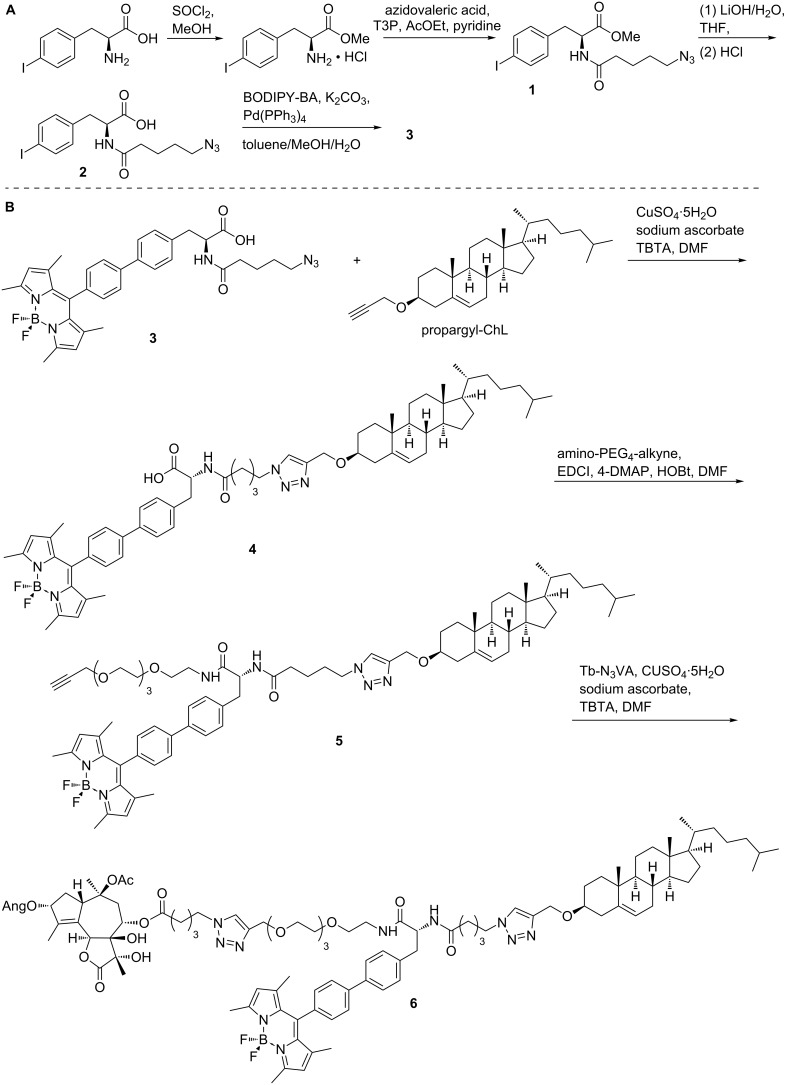
Synthesis of the BODIPY building block (part A) and construct **6** (part B).

In this work, we describe the synthesis and application of a fluorescent construct (further called construct **6**, depicted in Scheme 1) based on a green-emitting BODIPY dye and trilobolide–cholesterol (Tb-ChL) in a liposome formulation. Trilobolide (Tb, Figure 1) is a potent natural compound of the sesquiterpene lactone class, which causes cell death via depleting intracellular Ca^2+^ ion stores by the irreversible inhibition of sarco-/endoplasmic reticulum Ca^2+^-ATPase (SERCA) already at nanomolar concentrations [27–30]. In our recent study, we reported the localization of fluorescent Tb-BODIPY conjugates in the endoplasmic reticulum of a number of cancer cell lines [31]. Besides that, Tb is of high interest also for the fact that it induces high production of nitric monoxide (NO) which has an immunomodulating effect on rat peritoneal cells [32]. We documented in [31] that Tb, prepared as a fluorescent conjugate with green-emitting BODIPY, induced a dose-dependent NO production in primary rat macrophages. The potency of the fluorescent Tb to express inducible NO and cytokine secretion was shifted to a low micromolar range in comparison to the submicromolar activity of Tb itself.

The introduction of cholesterol (ChL) in the proposed structure is based on its routine exploitation in production of artificial liposome vehicles. Incorporation of ChL into liposomes was shown to ‘tighten’ the fluid bilayers, and thus, to reduce the leakage of an active content from the liposomes [1]. Taken together, a construct **6** probe, containing Tb, ChL and BODIPY, represents a well-defined traceable system with a potentiated ability to assemble into liposomal systems.

## Results and Discussion

### Chemistry

In this work, Tb was connected to a pegylated BODIPY building block containing ChL. This way obtained construct **6** was used as a component for liposomal formulation. The syntheses of some of the employed molecules were previously described [24,27–28], their structures are shown in Figure 1.

The synthesis of a BODIPY-based building block is displayed in Scheme 1, part A. Methyl 4-iodo-L-phenylalaninate hydrochloride was prepared by the reaction of 4-iodo-L-phenylalanine with thionyl chloride in MeOH in quantitative yield [33]. The successive acylation of the α-amino group with 5-azidovaleric acid catalyzed by T3P (propylphosphonic anhydride) in the mixture of pyridine and AcOEt gave azidoterminated product **1** in 70% yield. Alkaline hydrolysis of methyl ester **1** with aqueous LiOH in THF and subsequent Suzuki cross-coupling with BODIPY-BA [34] catalyzed by Pd(PPh_3_)_4_ and K_2_CO_3_ in a mixture of toluene/MeOH/water provided the fluorescent building block **3** in 88% yield.

Sequential connection of other functional components of the target compound is shown in Scheme 1, part B. Propargyl-ChL [35] was introduced into Huisgen copper-catalyzed 1,3-dipolar cycloaddition [36] (CuAAC) with BODIPY **3**. This microwave-assisted reaction catalyzed by CuSO_4_·5H_2_O, sodium ascorbate and a catalytic amount of TBTA (tris[(1-benzyl-1*H*-1,2,3-triazol-4-yl)methyl]amine) [37] in DMF gave a cholesterol-containing clickate **4** in 49% yield. The pegylation of **4** with amino-PEG_4_-alkyne in the presence of EDCI (*N*-(3-dimethylaminopropyl)-*N′*-ethylcarbodiimide hydrochloride), 4-DMAP (4-dimethylaminopyridine) and HOBt (*N*-hydroxybenzotriazole) in DMF provided an alkyne-terminated intermediate **5** in excellent yield (92%). Finally, CuAAC cycloadition of **5** and Tb-N_3_VA [31] gave the target fluorescent construct **6** in good yield (84%).

**Figure 2:**
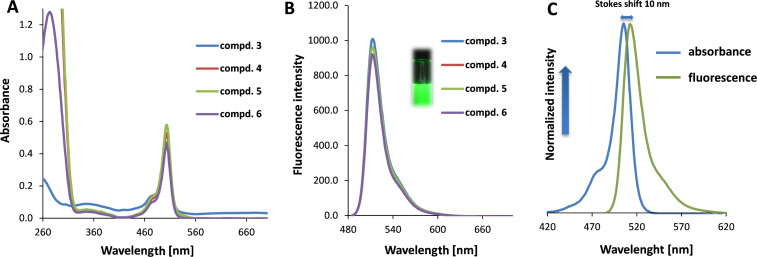
Absorbance and fluorescence spectra of compounds **3**–**6**. UV spectra (part A) were recorded with a concentration of 10 μM in DCM and fluorescence spectra (part B) with a concentration of 0.1 μM in DCM using an excitation wavelength of 475 nm. A typical Stokes shift (10 nm) is demonstrated for construct **6** (part C).

The absorbance and fluorescence emission spectra of compounds **3**–**6** are depicted in Figure 2.

Compounds **3**–**6** showed absorption and emission maxima at 503 and 513 nm (excitation at 475 nm), respectively. The molar extinction coefficients of **3**–**6** in DCM ranged from 45,000 to 58,000 L·mol^−1^·cm^−1^. The purity of the target construct **6** was determined by HPLC–MS and proved to be ≥95% (Supporting Information File 1, section 5.3, Figure S15). Thereafter, construct **6** was used for liposomal formulation and biological experiments, in which the immunomodulatory, delivery and anticancer potential was evalueated.

### Nitric oxide release in primary macrophages

**Figure 3:**
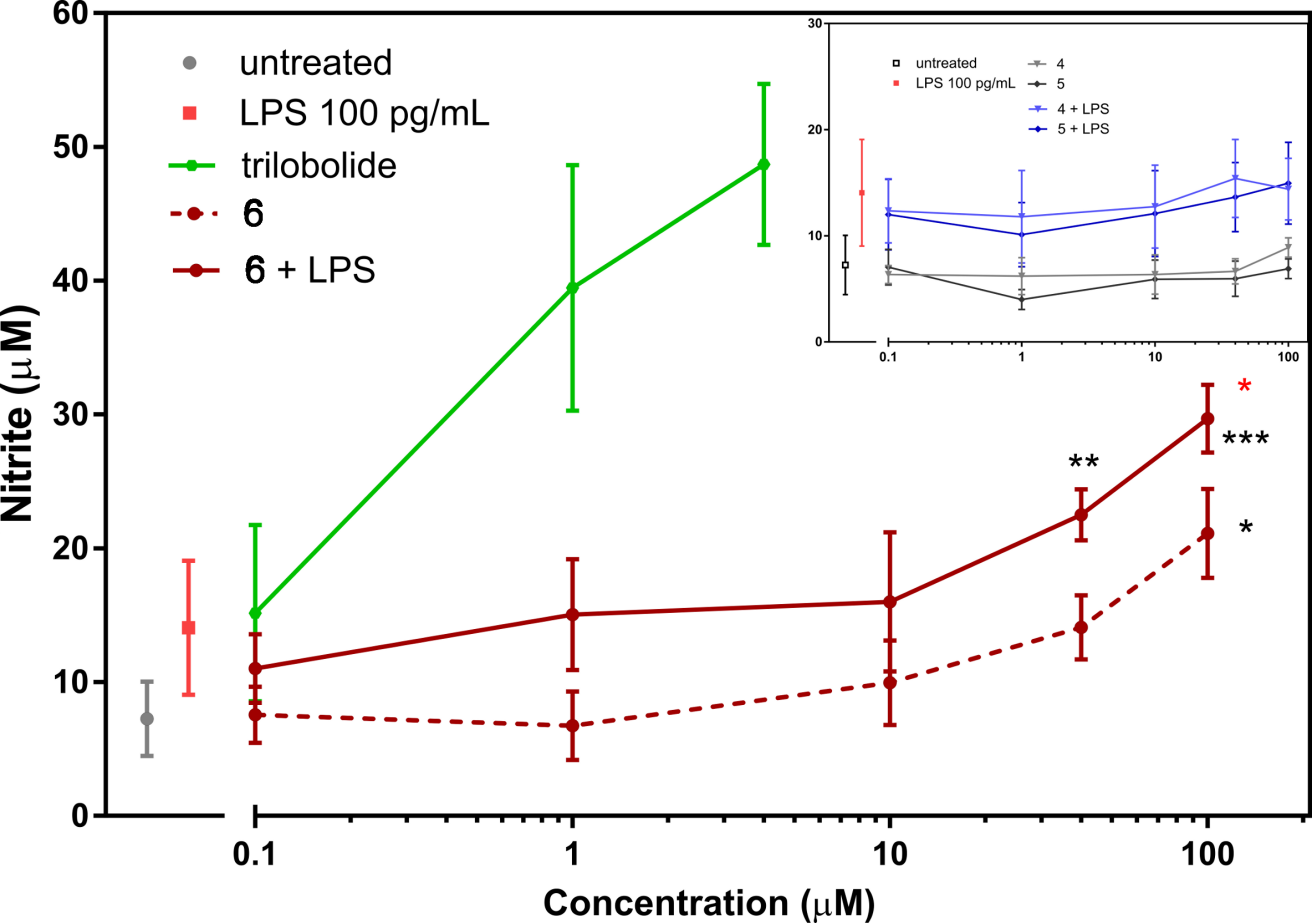
NO production in primary rat macrophages. The cells were treated with Tb, compounds **4**, **5**, and Tb-construct **6** for 24 h with or without lipopolysaccharide (LPS, 100 pg·mL^−1^). The results represent the mean ± SEM of 2 independent experiments, *n* = 4. Statistical significance: *P < 0.05, **P < 0.01, ***P < 0.001; black*: compounds are significantly different from untreated cells, red*: the compound is significantly different from LPS-treated cells.

NO (nitric oxide) is one of the most important effector molecules in the repertoire of non-specific immune defence mechanisms. This molecule is produced by macrophages and the antimicrobial and antiparasitic properties of NO have been well described [38]. Currently, the role of NO as a mediator between chronic inflammation and carcinogenesis is intensively studied [39]. The expression of inducible NO is under control of a number of cytokines. Alternatively, lipopolysaccharide (endotoxin) is known as strong inducer of NO in macrophages. Since it is known that sesquiterpene lactones, Tg, Tb, as well as Tb derivatives [31], possess strong stimulating activity for NO production by immune cells [40–41], we examined whether construct **6**, also based on Tb, exhibits similar immunobiological properties. The production of NO was evaluated after 24 h of cultivation of primary rat macrophages in the presence of increasing concentrations of Tb and construct **6**. In this study, we observed the typical activity of Tb to induce NO production in rodent macrophages which started below 0.1 µM Tb and reached an NO production of 50 µM in the presence of 4 µM Tb (the highest concentration tested, Figure 3).

The tested construct **6** induced moderate dose-dependent NO induction (methods in Supporting Information File 1, sections 4.3 and 4.4). The significant increase of NO to 21 µM was observed only at the highest concentration of 100 µM of construct **6** (*P < 0.05). We also investigated an eventual synergistic effect of construct **6** and lipopolysaccharide (LPS) in macrophage immunomodulation. To activate the macrophages, only a low concentration of an immunostimulator (LPS, 100 pg·mL^−1^) was used. In the presence of LPS, the dose-dependent curve for NO production was running higher and it was in parallel with the curve of non-stimulated cells. The synergistic effect of construct **6** with LPS on increased NO production was detected at 40 µM concentration of Tb-construct **6**, and it was significantly pronounced at 100 µM of construct **6** (*P < 0.05 vs LPS), upon which the level of NO reached 30 µM concentration. As expected, no effect on NO synthesis was found for **4** and **5** BODIPYs-ChL derivatives not containing Tb. No changes were detected in cell viability (WST-1 assay) for compounds **4**, **5**, and **6** (data not shown). From these and previous findings [30–31], we can summarize that the reduced immunomodulatory activity of Tb construct **6** is given by its high molecular weight (*M*_W_ equal to 1814) in comparison to Tb (*M*_W_ equal to 522), and overall shape of the molecule. Further, cholesterol is one of the basic natural components of eukaryotic cells, thus some portion of construct **6** could be fixed in plasma membrane, which decreases the possibility of manifesting the known biological effects of Tb inside cells [42].

### Liposome preparation and characterization

**Figure 4:**
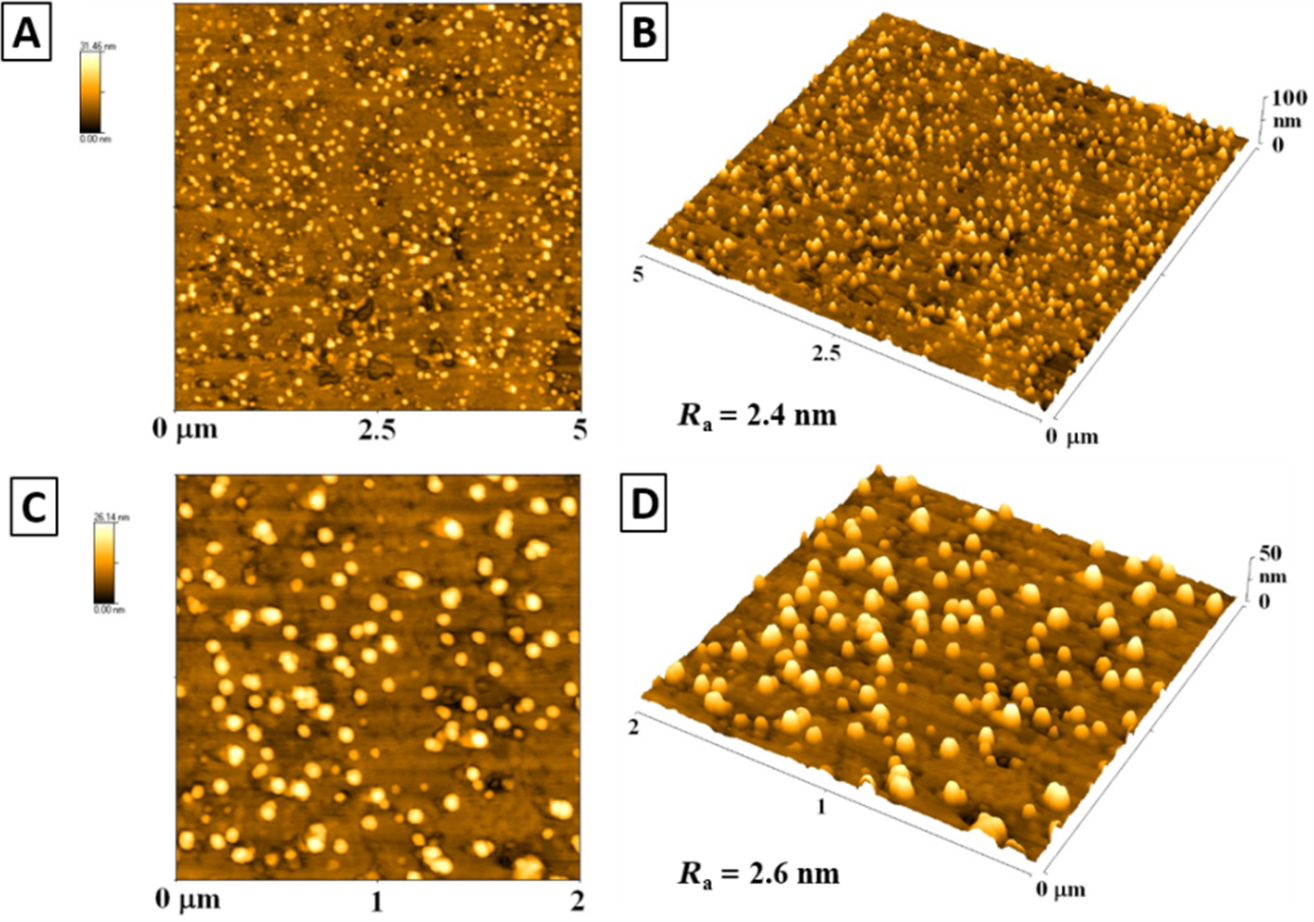
Atomic force microscopy images of liposomes, 5 µm area: A) 2D image, B) 3D image (*R*_a_ = 2.4 nm); 2 µm area: C) 2D image, D) 3D image (*R*_a_ = 2.6 nm). *R*_a_ represents the arithmetic average of the deviations from the centre plane of the sample.

Liposomes were prepared by a reverse-phase evaporation method followed by homogenization (Supporting Information File 1, section 2). Dipalmitoyl-3-trimethylammonium-propane (DPTAP), phosphatidylethanolamine (DOPE) and ChL were used for implementation of fluorescent construct **6** into liposomal formulation (ratio 4:4:1:1, respectively). A hydrophobic film prepared by evaporation of a lipid–chloroform solution was hydrated with physiological solution. The desired unilamellar vesicles were obtained by homogenization of the dispersion through a 100 nm pore size polycarbonate filter. Characterization of the prepared liposomes with incorporated construct **6** was performed by atomic force microscopy (AFM) analysis in a tapping mode in 2D and 3D arrangement, see Figure 4 (Supporting Information File 1, section 3).

We confirmed the successful preparation of liposomes, the average size of which was 150 nm in width and 30 nm in height. The larger dimension of the liposomes in width, than expected, was probably caused by their adhesion to the glass surface, which was on the other hand necessary in order to perform the AFM analysis. The values of average roughness described in the Figure 4 for both (2 × 2) and (5 × 5) μm^2^ are almost similar, therefore the uniformity of prepared liposomes over the surface was proven (no significant differences caused by change in the surface structure). It was confirmed, on the basis of surface roughness for both scanning areas and the evaluation of height and width of globular structures, that prepared liposomes were uniform in shape (high variability in shape or inhomogeneous peak structure would extensively increase the roughness value) and the cover over the surface was also homogeneous.

### Live-cell imaging of construct **6** and its liposomal formulation

The potency of the fluorescent construct Tb-ChL and BODIPY and its liposome derivative to enter cancer cells was tested by live-cell fluorescence microscopy using two human cell line models: cells were derived from osteosarcoma (U-2 OS) and cervical carcinoma (HeLa).

**Figure 5:**
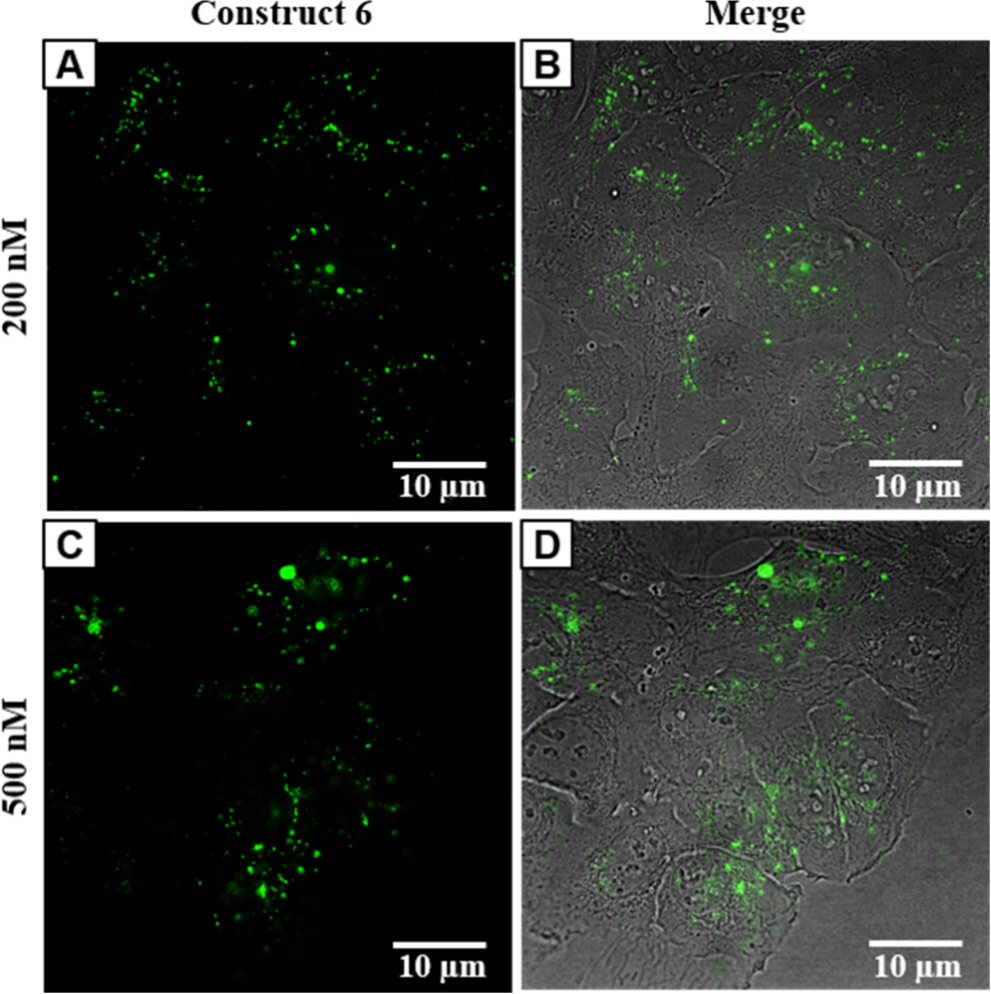
Panel of images from live-cell fluorescence microscopy: intracellular localization of construct **6** in U-2 OS cells after 48 h of incubation: A) 200 nM; C) 500 nM concentration of construct **6**; B) and D) merges of A and C with bright field images of U-2 OS cells, respectively.

Inside U-2 OS cells, construct **6** was localized from 200 nM concentration already after 1 h of incubation, the fluorescent signal was of dot-like character and persisted for at least 48 h until which, the intracellular localization was followed (Figure 5).

A similar situation was observed in HeLa cells (Supporting Information File 1, Figure S17), in which the construct **6** was also internalized and its distribution resembled the structure of the endoplasmic reticulum as well as partially the cell membrane.

**Figure 6:**
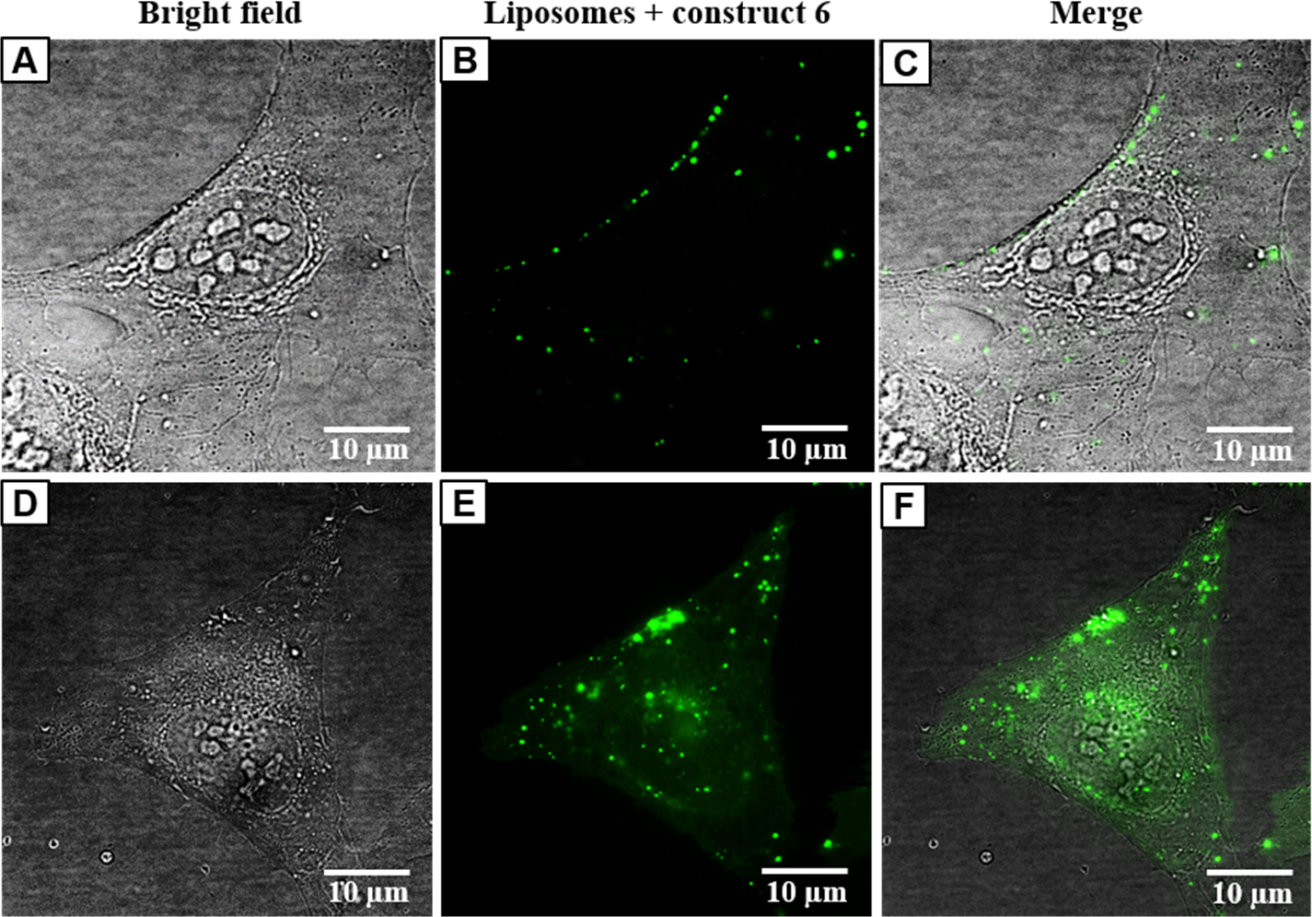
Panel of images from live-cell fluorescence microscopy: intracellular localization of liposomes with construct **6** (250 nM) in U-2 OS cells after 2 h of incubation: A, D) bright field; B, E) construct **6** in liposomes; C, F) merged images of A and B and D and E.

In the case of liposomes containing construct **6**, intracellular uptake was detected already at 43 nM concentration after 1 h of incubation with U-2 OS cells (Supporting Information File 1, Figure S16), on which they were bound at the plasma membrane. After 2 h of incubation, there were two populations of cells with liposomes bound either on the plasma membrane or inside the cells (Figure 6).

The intracellular localization of liposomes was further pronounced with increased concentration up to 1.25 µM (Supporting Information File 1, Figure S18) after 2 h of incubation. With prolonged time (7 h), the U-2 OS cells were disrupted and underwent cell death (Supporting Information File 1, Figure S18), which could be caused by the release of the active construct **6** from the liposomes. Further tests are necessary to confirm this hypothesis.

## Conclusion

In summary, in order to develop a drug delivery system for potential theranostic applications, we prepared a submicron liposome-based formulation of a cytotoxic agent, sesquiterpene lactone, trilobolide. More specifically, we synthesized and characterized a fluorescent construct of Tb conjugated to cholesterol and a green-emitting BODIPY dye, which was successfully incorporated into liposomes. The immunomodulatory activity tested in primary rat macrophages revealed significant dose-dependent NO production in the presence of LPS; at 100 µM concentration of construct **6**, the level of NO raised up to 30 µM. In further biological evaluation, we found that construct **6** was efficiently localized inside human U-2 OS and HeLa cancer cells. The encapsulation of construct **6** into liposomes resulted in sufficient distribution inside the cancer cells. The intracellular trafficking pattern of liposomes was characterized by two populations: the first one clearly localized on the cell membrane and the other inside the cells. With prolonged time, the population with internalized liposomes was linked to cell death, which might be caused by the release of active construct **6** from liposomes in cells. This study could be useful for further design and optimization of analogous systems for theranostic liposomal drug-delivery applications.

## Supporting Information

File 1 Additional information, characterization methods, experimental, analytical data, and supporting images from live-cell fluorescence microscopy.
