# Supplementary material for: BODIPY-based fluorescent liposomes with sesquiterpene lactone trilobolide
Source: Beilstein J Org Chem. 2017 Jul 4;13:1316–24. doi: 10.3762/bjoc.13.128 (PMC5530629; doi:10.3762/bjoc.13.128)
Supplement: File 1 — Additional information, characterization methods, experimental, analytical data, and supporting images from live-cell fluorescence microscopy. [file Beilstein_J_Org_Chem-13-1316-s001.pdf]

Supporting Information

for

BODIPY-based fluorescent liposomes with sesquiterpene lactone trilobolide

Ludmila Škorpilová[1,2], Silvie Rimpelová[3], Michal Jurášek[1], Miloš Buděšínský[4], Jana Lokajová[4], Roman Effenberg[1], Petr Slepička[5], Tomáš Ruml[3], Eva Kmoníčková[6,7], Pavel B. Drašar[1,]*, and Zdeněk Wimmer[1,2]*

Address: [1]Department of Chemistry of Natural Compounds, University of Chemistry and Technology Prague, Technická 5, 166 28 Prague 6, Czech Republic, [2]Institute of Experimental Botany, ASCR, Vídeňská 1083, 142 20 Prague 4, Czech Republic, [3]Department of Biochemistry and Microbiology, University of Chemistry and Technology Prague, Technická 5, 166 28 Prague 6, Czech Republic, [4]Institute of Organic Chemistry and Biochemistry, ASCR, Flemingovo n. 2, 166 10 Prague 6, Czech Republic, [5]Department of Solid State Engineering, University of Chemistry and Technology Prague, Technická 5, 166 28 Prague 6, Czech Republic, [6]Institute of Experimental Medicine, ASCR, Vídeňská 1083, 142 20 Prague 4, Czech Republic and [7]Charles University, Faculty of Medicine in Pilsen, Alej Svobody 76, 323 00 Pilsen, Czech Republic

* Corresponding author

Email: Pavel B. Drašar - pavel.drasar@vscht.cz; Zdeněk Wimmer - wimmer@biomed.cas.cz

Additional information, characterization methods, experimental, analytical data, and supporting images from live-cell fluorescence microscopy

Table of Contents

**1.**	**Chemistry**	S3
1.1.	Methods and apparatus	S3
1.2.	Synthesis	S3
	Methyl *N* -(5-azidopentanoyl)-4-iodo-L-phenylalaninate (**1**)	S3
	4-(BODIPY)-*N*-(5-azidopentanoyl)phenylalanine (**3**)	S4
	ChL-BODIPY-acid (**4**)	S5
	ChL-BODIPY alkyne (**5**)	S6
	Tb-BODIPY-ChL construct (**6**)	S7
**2.**	**Liposome preparation**	S9
**3.**	**Atomic force microscopy**	S10
**4.**	**Cell assays**	S11
4.1.	Cell uptake of Tb-BODIPY-ChL construct **6**	S11
4.2.	Fluorescence microscopy	S11
4.3	Nitric oxide release in primary macrophages	S12
4.4.	Statistical analysis	S12
**5.**	**Analytical supplement**	S13
5.1.	Graphical assignment of the ^{1}H and ^{13}C NMR spectra of the key compounds	S13
5.2.	High-resolution mass spectra	S18
5.3.	HPLC chromatogram of the target construct **6**	S20
**6.**	**Supplementary live-cell images**	S21

1. Chemistry

1.1. Methods and apparatus

Cholesterol was purchased from Sigma-Aldrich (Corp., St. Louis, USA), acetylene-PEG$_3$-amine from Click chemistry tools (Scottsdale, USA). For thin-layer chromatography (TLC), aluminum silica gel sheets were used for detection in UV light (TLC Silica gel 60 F$_{254,}$ Merck). A diluted solution of sulfuric acid in methanol was used and plates were successively heated for TLC visualization. Silica gel (30-60 µm, SiliTech, MP Biomedicals) was used for column chromatography. NMR spectra (^{1}H 300 MHz and ^{13}C 75 MHz) were recorded with a Varian Gemini 300 (Varian, Palo Alto, USA). Chemical shifts are given in δ (ppm). FTIR spectra were measured by Nicolet iS10 (Thermo Scientific, Waltham, USA) using ATR (KBr crystal) technique (symbolism; *s* strong, *vs* very strong, *m* medium, *w* weak, *vw* very weak). HRMS were measured by LTQ ORBITRAP VELOS with HESI$^+$/HESI$^-$ ionization (Thermo Scientific, Waltham, USA). Optical rotations were measured by an Autopol VI polarimeter (Rudolph Research Analytical, Hackettstown, USA). Methyl 4-iodo-L-phenylalaninate, propargyl cholesteryl ether [1], Tb-N$_3$VA [2], and BODIPY-BA [3] were synthesized according to previously described protocols.

1.2. Synthesis

Methyl *N*-(5-azidopentanoyl)-4-iodo-L-phenylalaninate (**1**)

Pyridine (15 mL) and T3P (6 mL, 0.02 mol, 50% solution) were added to a solution of methyl 4-iodo-L-phenylalaninate hydrochloride (1.5 g, 4.9 mmol) and azidovaleric acid (0.85 g, 5.9 mmol) in AcOEt (10 mL) at 0 °C. Then, the mixture was stirred at rt for 24 h. After the completition of the reaction, the mixture was dilluted with AcOEt (50 mL), the organic layer was washed with brine (3 × 50 mL), dried over $MgSO_4$, filtered and the solvent was evaporated under reduced pressure. The product was purified by column chromatography

on silica gel using a gradient mobile phase (DCM/MeOH 100:0 to 98:2). Evaporation of the solvent yielded a white-yellow product (1.6 g, 3.7 mmol) in 70% yield. ^{1}H NMR (300 MHz, $CDCl_3$) δ ppm: 7.54 (d, *J* = 8.2 Hz, 2 H), 6.80 (d, *J* = 8.2 Hz, 2 H), 6.29 (d, *J* = 7.9 Hz, 1 H), 4.87 - 4.73 (m, 1 H), 3.66 (s, 3 H), 3.19 (t, *J* = 6.6 Hz, 2 H), 3.08 - 2.98 (m, 1 H), 2.95 - 2.84 (m, 1 H), 2.13 (t, *J* = 7.0 Hz, 2 H), 1.65 - 1.53 (m, 2 H), 1.53 - 1.41 (m, 2 H). ^{13}C NMR (75MHz, $CDCl_3$) δ ppm: 172.4, 172.2, 137.8, 135.9, 131.5, 92.8, 53.0, 52.7, 51.3, 37.6, 35.7, 28.4, 22.8. LRMS-ESI: found 306.0 $[M+H]^+$ and 324.0 $[M+NH_4]^+$.

4-(BODIPY)-*N*-(5-azidopentanoyl)phenylalanine (**3**)

A solution of LiOH (166 mg) in water (8 mL) was added to a solution of methyl ester (**1**, 1 g, 2.3 mmol) in THF (16 mL), and the mixture was stirred for 3 h. Then, the pH value was changed to acidic by HCl, and the product was extracted by AcOEt (5 × 25 mL). The organic layers were combined, washed with brine (2 × 100 mL) and dried over $MgSO_4$. The solvents were evaporated under reduced pressure. The product **2** was used in the following reaction without purification (LRMS-ESI: 415.0 $[M-H]^-$). The acid **2** (388 mg, 0.93 mmol) and BODIPY-BA [3] (480 mg, 1.3 mmol) were dissolved in a mixture of toluene/methanol/water (10:3:2; 15 mL). Potassium carbonate (1.28 g, 9.3 mmol) and $Pd(PPh_3)_4$ (108 mg, 0.1 mmol) were added, and the mixture was heated at 60 °C for 24 h. Chloroform (30 mL) was added, and the mixture was filtered. The organic layer was washed with diluted HCl (50 mL), water (50 mL) and a 1 M solution of ammonium chloride (50 mL), then dried over $MgSO_4$, filtered and the solvent was evaporated under reduced pressure. The product was purified by column chromatography on silica gel using a gradient mobile phase (DCM/MeOH 100:0 to 98:2). Evaporation of the solvent yielded a product as brick solids (500 mg, 0.82 mmol) in 88% yield.. ^{1}H NMR (600 MHz, $CDCl_3$+CD_3OD 2 drops): δ ppm 7.74 (m, 2H), 7.62 (m, 2H), 7.35 (m, 2H), 7.30 (m, 2H), 6.01 (br s, 2H), 4.85 (dd, 1H, *J* = 7.0, 5.4 Hz), 3.30 (dd, 1H, *J* = 14.0,

5.4 Hz), 3.23 (m, 2H), 3.11 (dd, 1H, J = 14.0, 7.0 Hz), 2.56 (s, 6H), 2.23 (m, 2H), 1.66 (m, 2H), 1.54 (m, 2H), 1.45 (s, 6H). ^{13}C NMR (150.9 MHz), $CDCl_3$+CD_3OD 2 drops): δ ppm 173.50, 173.07, 155.50, 143.32, 141.61, 141.30, 138.60, 136.21, 133.84, 131.47, 129.94, 128.51, 127.47, 127.05, 121.33, 53.05, 51.08, 37.13, 35.50, 28.18, 22.70, 14.48. For graphical assignment of the ^{1}H and ^{13}C NMR, see section 5.1 - Figure S1. IR ($CHCl_3$, neat): 3285 (*s*), 3071 (*w*), 2952 (*m*), 2862 (*w*), 2095 (*s*), 1736 (*vs*), 1648 (*s*), 1541 (*s*), 1485 (*w-m*), 1436 (*m*), 1351 (*w-m*), 1272 (*m*), 1260 (*m-s*), 1219 (*m*), 1173 (*w*), 1009 (*w*), 817 (*w*). HRMS-ESI: *monoisotopic mass* 612.2832 Da, found *m/z* 611.2827 $[M-H]^-$; section 5.2 - Figure S11. ε = 47 000 $dm^3 \cdot mol^{-1} \cdot cm^{-1}$. $[\alpha]_D^{20}$ +11 (c = 1; $CHCl_3$).

ChL-BODIPY-acid (**4**)

Procedure for CuAAC: To a solution of 3-*O*-propargylcholesterylether (83 mg, 0.2 mmol) and BODIPY derivative **3** (100 mg, 0.16 mmol) in DMF (2 mL) were added $CuSO_4 \cdot 5H_2O$ (4 mg, 0.02 mmol), sodium ascorbate (6.5 mg, 0.03 mmol) and TBTA (17 mg, 0.03 mmol). The mixture was placed onto a microwave reactor (MW) and stirred at 60 °C for 90 min. The solvent was evaporated and the product was purified by column chromatography on silica gel (DCM/MeOH 100:0 to 98:2) to obtain product **4** as a brick coloured gel (82 mg, 0.08 mmol) in 49% yield. ^{1}H NMR (600 MHz, $CDCl_3$): δ ppm: 7.69 (m, 2H), 7.57 (m, 2H), 7.32 (m, 2H), 7.26 (m, 2H), 7.25 (s, 1H), 6.38 (br s, 1H), 5.98 (br s, 2H), 5.34 (m, 1H), 4.91 (m, 1H), 4.68 (br s, 2H), 4.33 (m, 2H), 3.35 (tt, 1H, J = 11.0, 11.0, 4.5, 4.5 Hz), 3.24 (dd, 1H, J = 14.0, 5.0 Hz), 3.06 (dd, 1H, J = 14.0, 7.0 Hz), 2.56 (s, 6H), 2.41 (m, 1H), 2.26 (m, 1H), 2.22 (m, 2H), 2.00 (dt, 1H, J = 13.0, 3.5, 3.5 Hz), 1.96 (m, 2H), 1.88 (m, 2H), 1.85 (m, 1H), 1.82 (m, 1H), 1.63 (m, 1H), 1.57 (m, 1H), 1.56 (m, 1H), 1.52 (m, 2H), 1.50 (m, 1H), 1.46 (m, 2H), 1.43 (m, 1H), 1.41 (s, 6H), 1.36 (m, 1H), 1.34 (m, 1H), 1.33 (m, 1H), 1.25 (m, 1H), 1.13 (m, 4H), 1.08 (m, 1H), 1.06 (m, 1H), 1.04 (m, 1H), 0.99 (m, 1H), 0.988 (s, 3H), 0.97 (m, 1H), 0.91 (m, 1H),

0.908 (d, 3H, *J* = 6.5 Hz), 0.865 (d, 3H, *J* = 6.5 Hz), 0.86 (d, 3H, *J* = 6.5 Hz), 0.664 (s, 3H); Fig. S5. ^{13}C NMR (150.9 MHz, $CDCl_3$) *δ* ppm: 173.33, 172.56, 155.52, 142.99, 141.35, 141.03, 140.43, 138.55, 135.92, 133.96, 131.40, 130.00, 128.52, 127.35, 127.06, 122.02, 121.24, 79.41, 61.46, 56.71, 56.14, 53.07, 50.10, 49.81, 42.29, 39.73, 39.49, 38.92, 37.10, 36.81, 36.16, 35.76, 35.14, 31.91, 31.85, 29.15, 28.20, 28.00, 24.26, 23.83, 22.80, 22.54, 22.57, 21.04, 19.33, 18.69, 14.59, 14.54, 11.84; Fig. S6. For graphical assignment of the ^{1}H and ^{13}C NMR, see section 5.1 - Figure S2. IR ($CHCl_3$, neat): 3287 (*br*), 3028 (*w*), 2931 (*m*), 2866 (*m*), 2360 (*m*), 2340 (*m*), 1958 (*w*), 1919 (*w*), 1844 (*w*), 1771 (*w*), 1734 (*m*), 1669 (*m*), 1636 (*m*), 1544 (*s*), 1510 (*s*), 1468 (*m*), 1308 (*m*), 1194 (*s*), 1157 (*m*), 1085 (*m*), 981 (*m*), 817 (*w*). HRMS-ESI: *monoisotopic mass* 1036.6537 Da, 1059.64465 $[M+Na]^+$; section 5.2 - Figure S12. ε = 53 000 $dm^3 \cdot mol^{-1} \cdot cm^{-1}$. $[\alpha]_D^{20}$ + 5.33 (*c* = 1; $CHCl_3$).

ChL-BODIPY alkyne (**5**)

EDCI (16.6 mg, 0.09 mmol), 4-DMAP (10.6 mg, 0.09 mmol) and HOBt (3.9 mg, 0.03 mmol) were added sequentially to a solution of **4** (60 mg, 0.06 mmol) and amino-PEG$_4$-acetylene (19 mg, 0.08 mmol) in DMF (5 mL) at 0 °C. The cooling bath was removed and the mixture was stirred for 24 h. Additional EDCI (5.5 mg, 0.03 mmol) was added, and stirring at rt continued for the next 24 h. The solvent was evaporated under reduced pressure, and the product was purified by column chromatography on a silica gel (DCM/MeOH 100:0 to 98:2) to obtain product **5** as a brick like gel (66 mg, 0.05 mmol) in 92% yield. ^{1}H NMR (600 MHz, $CDCl_3$) *δ* ppm: 7.73 (m, 2H), 7.61 (m, 2H), 7.54 (s, 1H), 7.345 (m, 2H), 7.31 (m, 2H), 6.62 (t, 1H, *J* = 5.2 Hz), 6.42 (d, 1H, *J* = 8.0 Hz), 5.99 (br s, 2H), 5.35 (m, 1H), 4.70 (ddd, 1H; *J* = 8.0, 7.0, 6.2 Hz), 4.67 (br s, 2H), 4.31 (m, 2H), 4.20 (d, 1H, *J* = 2.2 Hz), 3.69 (m, 2H), 3.68–3.44 (m, 12H), 3.42 (m, 2H), 3.32 (tt, 1H, *J* = 11.2, 11.2, 4.5, 4.5 Hz), 3.14 (dd, 1H, *J* = 13.6, 7.0 Hz), 3.09 (dd, 1H, *J* = 13.6, 6.2 Hz), 2.56 (s, 6H), 2.44 (t, 1H, *J* = 2.2 Hz), 2.41 (ddd, 1H,

J = 13.0, 4.5, 2.2 Hz), 2.25 (m, 1H), 2.23 (m, 2H), 2.00 (m, 1H), 1.965 (m, 1H), 1.95 (m, 1H), 1.90 (m, 2H), 1.85 (m, 1H), 1.825 (m, 1H), 1.65 (m, 2H), 1.57 (m, 1H), 1.515 (m, 2H), 1.495 (m, 1H), 1.49 (m, 1H), 1.44 (m, 1H), 1.43 (m, 1H), 1.43 (s, 6H), 1.37 (m, 1H), 1.33 (m, 2H), 1.255 (m, 1H), 1.15 (m, 1H), 1.14 (m, 1H), 1.12 (m, 2H), 1.085 (m, 1H), 1.06 (m, 1H), 1.04 (m, 1H), 0.994 (s, 3H), 0.99 (m, 1H), 0.98 (m, 1H), 0.915 (m, 1H), 0.912 (d, 3H, J = 6.6 Hz), 0.866 (d, 3H, J = 6.6 Hz), 0.862 (d, 3H, J = 6.6 Hz), 0.672 (s, 3H); Fig. 7. ^{13}C NMR (150.9 MHz, $CDCl_3$) δ ppm: 171.70, 170.60, 155.48, 145.92, 143.00, 141.37, 141.07, 140.61, 138.40, 136.44, 133.91, 131.40, 129.98, 128.50, 127.35, 127.01, 122.22, 121.80, 121.21, 79.60, 78.94, 74.72, 70.51, 70.49, 70.44, 70.29, 70.15, 69.51, 69.02, 61.62, 58.36, 56.70, 56.10, 54.22, 50.10, 49.86, 42.27, 39.72, 39.47, 39.30, 38.99, 38.46, 37.11, 36.80, 36.14, 35.74, 35.31, 31.89, 31.84, 29.52, 28.27, 28.19, 27.97, 24.24, 23.78, 22.78, 22.52, 22.23, 21.02, 19.32, 18.67, 14.56, 14.52, 11.82; Fig. S8. For graphical assignment of the ^{1}H and ^{13}C NMR, see section 5.1 - Figure S3. IR ($CHCl_3$, neat): 3284 (*br*), 3072 (*w*), 2932 (*m*), 2867 (*m*), 2360 (*w*), 2113 (*w*), 1958 (*w*), 1639 (*m*), 1544 (*s*), 1511 (*m*), 1468 (*m*), 1366 (*m*), 1307 (*m*), 1261 (*s*), 1195 (*s*), 1089 (*s*), 981 (*m*), 836 (*w*), 711 (*w*). HRMS-ESI: *monoisotopic mass* 1249.7902 Da, found *m/z* 1250.79895 $[M+H]^+$, 1272.78075 $[M+Na]^+$ and 1288.75415 $[M+K]^+$; section 5.2 - Figure S13. ε = 58 000 $dm^3 \cdot mol^{-1} \cdot cm^{-1}$. $[\alpha]_D^{20}$ – 10.3 (c = 1, $CHCl_3$).

Tb-BODIPY-ChL construct (**6**)

This compound was synthesized by following the click protocol described for compound **4**. In reaction: Tb-N_3VA (15.5 mg, 0.03 mmol), ChL-BODIPY alkyne **5** (35 mg, 0.03 mmol), $CuSO_4 \cdot 5H_2O$ (0.2 equiv), sodium ascorbate (0.3 equiv) and TBTA (0.2 equiv), DMF (2 mL), the mixture was placed onto MW and stirred at 60 °C for 5 h. Chromatography on silica gel (2% of MeOH in DCM) gave product **6** (42 mg, 0.023 mmol) in the yield of 84% as a brick coloured gel which solidified upon storage. ^{1}H NMR (600 MHz), $CDCl_3$) δ ppm:

7.72 (m, 2H), 7.64 (s, 1H), 7.575 (m, 2H), 7.54 (s, 1H), 7.34 (m, 2H), 7.29 (br, 1H), 7.28 (m, 2H), 6.92 (d, 1H, *J* = 8.0 Hz), 6.11 (qq, 1H, *J* = 7.2, 1.5 Hz), 5.99 (br s, 2H), 5.80 (t, 1H, *J* = 3.8 Hz), 5.75 (q, 1H, *J* = 1.8 Hz), 5.60 (m, 1H), 5.34 (m, 1H), 4.71 (dt, 1H; *J* = 8.0, 7.0, 7.0 Hz), 4.685 (d, 1H, *J* = 12.0 Hz), 4.65 (d, 1H, *J* = 12.0 Hz), 4.64 (br s, 2H), 4.34 (m, 1H), 4.32 (m, 3H), 4.27 (m, 1H), 3.71 (m, 2H), 3.66–3.59 (m, 8H), 3.52 (m, 1H), 3.47 (m, 1H), 3.46 (m, 1H), 3.38 (m, 1H), 3.30 (tt, 1H, *J* = 11.2, 11.2, 4.5, 4.5 Hz), 3.28 (m, 2H), 3.055 (dd, 1H, *J* = 13.5, 7.0 Hz), 3.00 (dd, 1H, *J* = 13.5, 7.0 Hz), 2.98 (dd, 1H, *J* = 14.6, 3.8 Hz), 2.59 (m, 1H), 2.56 (s, 6H), 2.39 (m, 1H), 2.36 (dd, 1H, *J* = 14.6, 3.8 Hz), 2.34 (m, 2H), 2.27 (m, 1H), 2.23 (m, 1H), 2.19 (m, 1H), 2.02 (dq, 3H, *J* = 7.2, 1.5 Hz), 2.00 (m, 1H), 1.96 (m, 1H), 1.94 (m, 1H), 1.94 (s, 3H), 1.93 (m, 2H), 1.92 (p, 3H, *J* = 4 × 1.5 Hz), 1.92 (m, 3H), 1.88 (m, 2H), 1.85 (m, 1H), 1.82 (m, 1H), 1.66 (m, 1H), 1.61 (m, 2H), 1.58 (m, 2H), 1.56 (m, 1H), 1.51 (m, 2H), 1.49 (m, 1H), 1.48 (m, 1H), 1.44 (m, 1H), 1.425 (m, 1H), 1.425 (s, 6H), 1.405 (s, 3H), 1.35 (s, 3H), 1.335 (m, 1H), 1.33 (m, 1H), 1.25 (m, 1H), 1.15 (m, 1H), 1.135 (m, 1H), 1.12 (m, 2H), 1.08 (m, 1H), 1.06 (m, 1H), 1.04 (m, 1H), 0.99 (s, 3H), 0.99 (m, 1H), 0.975 (m, 1H), 0.92 (m, 1H), 0.92 (s, 3H), 0.866 (d, 3H, *J* = 6.6 Hz), 0.861 (d, 3H, *J* = 6.6 Hz), 0.67 (s, 3H); Fig. S9. ^{13}C NMR (150.9 MHz, $CDCl_3$) δ ppm: 176.42, 172.46, 171.85, 171.01, 170.33, 167.70, 155.49, 145.90, 144.98, 143.00, 142.74, 141.39, 141.12, 140.52, 138.31, 138.23, 136.49, 133.88, 132.05, 131.40, 130.02, 128.52, 127.83, 127.33, 126.88, 122.88, 122.48, 121.88, 121.22, 85.70, 79.69, 79.13, 78.65, 78.58, 77.63, 70.44, 70.41, 70.04, 69.79, 69.33, 66.69, 64.66, 61.47, 56.70, 56.11, 54.28, 51.68, 50.08, 49.81, 42.28, 39.72, 39.48, 39.32, 38.99, 38.58, 38.28, 37.09, 36.79, 36.15, 35.75, 35.39, 33.46, 32.37, 31.89, 31.84, 29.35, 29.29, 28.25, 28.20, 27.98, 24.25, 23.79, 22.79, 22.53, 22.37, 21.38, 21.16, 21.02, 20.70, 19.32, 18.68, 15.98, 14.57, 14.53, 13.13, 11.82; Fig. S10. For graphical assignment of the ^{1}H and ^{13}C NMR, see section 5.1 - Figure S4. IR ($CHCl_3$, neat): 3289 (*br*), 2927 (*s*), 2857 (*m*), 2367 (*w*), 2247 (*w*), 1958 (*w*), 1784 (*m*), 1731 (*m*), 1545 (*m*), 1465 (*m*), 1368 (*m*), 1247 (*m*), 1195 (*m-s*),

1156 (*s*), 1086 (*m-s*), 1050 (*m*), 982 (*m*), 838 (*w*), 732 (*w*), 609 (*w*). HRMS-ESI: *monoisotopic mass* 1814.0414 Da, found *m/z* 1815.05198 $[M+H]^+$, 1837.03136 $[M+Na]^+$ and 1853.00736 $[M+K]^+$; section 5.2 - Figure S14. ε = 45 000 $dm^3 \cdot mol^{-1} \cdot cm^{-1}$. $[\alpha]_D^{20}$ – 99 (*c* = 1, $CHCl_3$).

2. Liposome preparation

Liposomes were prepared by mixing stock solutions of construct **6** (1 $mg \cdot mL^{-1}$), 1,2-dipalmitoyl-3-trimethylammoniumpropane (DPTAP; 5 $mg \cdot mL^{-1}$), 1,2-dioleoyl-*sn*-glycero-3-phosphoethanolamine (DOPE; 2 $mg \cdot mL^{-1}$) and cholesterol (1 $mg \cdot mL^{-1}$) to yield a final concentration of compound **6** of 0.1 $mg \cdot mL^{-1}$ (55.13 µM). The appropriate mixture ratios of the stock solutions in chloroform were 10:10:40:40 (wt %) of construct **6**/DPTAP/DOPE/cholesterol. The resulting mixture was evaporated to dryness under a stream of pressurized air, and solvent residues were removed by evacuation under reduced pressure (8–100 mbar) for at least 16 h. The lipid residues were hydrated in physiological solution at 60 °C for 60 min with shaking to yield multilamellar vesicles. The resulting dispersion was processed to give large unilamellar vesicles by extruding the mixture 19 times through Millipore (Bedford, MA, USA) 100 nm pore size polycarbonate filters using a Liposo-Fast extruder. The liposome composition is depicted in Table S1.

Table S1. Composition of liposomes with incorporated construct **6**

**Component**	**wt [%]**	***c* [µg·mL^{-1}]**
Construct **6**	10	100
Cholesterol	10	100
1,2-Dipalmitoyl-3-trimethylammonium-propane	40	400
1,2-Dioleoyl-*sn*-Glycero-3-phosphoethanolamine	40	400

3. Atomic force microscopy

Atomic force microscopy (AFM) (instrument VEECO CP II, tapping mode, Si probe RTESPA-CP with the spring constant 20–80 N·m^{-1}) was used as the main method for characterization of the prepared liposomes by evaluating their surface morphology and roughness. For this purpose, the liposome samples (1 mg·mL^{-1}) with construct **6** were loaded (4 and 8 µL) on a microscopic glass coverslip, let semi-dry overnight and subjected to AFM analysis. The mean roughness value (R_a) represents the average of the deviations from the center plane of a sample. The R_a determination is calculated based on the following equation:

$$R_a = \frac{\sum_{i=1}^{N} \left| Z_i - Z_{cp} \right|}{N}$$

where Z_{cp} is the value of the central plane, Z_i is the actual Z value, and N is the number of points, where the Z_i is evaluated.

4. Cell assays

4.1. Cell uptake of Tb-BODIPY-ChL construct 6

In our study, the following human cancer cell lines were used: U-2 OS (cells from osteosarcoma) and HeLa (cells from cervical carcinoma). Cells were cultivated in DMEM medium (Sigma-Aldrich, USA) with stable L-glutamine, supplemented with 10% fetal bovine serum (Thermo Scientific, USA), and maintained at 37 °C in humidified atmosphere with 5% of CO_2.

Cells ($1 \cdot 10^5$ per dish) were seeded into 35 mm cultivation dishes with a glass bottom for live-cell imaging (Ibidi, Germany) and left to adhere for 16 h. The attached cells were washed with phosphate buffered saline (PBS, pH 7.4, 37 °C) and incubated with construct **6** (13–500 nM) and liposomes with encapsulated construct **6** at a concentration range of 13 nM–1.25 µM, in complete medium without phenol red (FluoroBrite DMEM; Thermo Scientific) at 37 °C for 1, 2, 7, 16 h, and 48 h, respectively. After the incubation period, the cells were washed with PBS and fed with fresh medium without phenol red and subjected to fluorescence microscopy.

4.2. Fluorescence microscopy

Cell uptake and the intracellular localization of Tb-BODIPY-ChL construct **6** and the liposome formulation were studied by real-time live-cell fluorescence microscopy in the subjected cell lines at 37 °C and in 5% CO_2 atmosphere. The images were acquired by an inverse fluorescence microscope Olympus IX-81, xCellence System using high-stability 150 W xenon arc burner, FITC filter (Olympus, Japan) and EM-CCD camera C9100-02 (Hamamatsu, Germany). Living cells were analyzed by a 60× oil immersion objective (NA 1.4, Olympus, Japan). All images were deconvolved using xCellence software.

4.3. Nitric oxide release in primary macrophages

The resident peritoneal macrophages collected from individual female Wistar rats weighing 175–185 g (VELAZ, Czech Republic; approved institutional protocol MSMT-15894/2013-310) were washed, resuspended and seeded into 96-well round-bottom microplates ($2{\cdot}10^5$ cells per well) in complete RPMI-1640 medium. The complete RPMI-1640 culture medium contained 10% heat-inactivated fetal bovine serum, 2 mM L-glutamine, 50 $\mu g{\cdot}mL^{-1}$ gentamicin, and $5{\cdot}10^{-5}$ M 2-mercaptoethanol (all Sigma-Aldrich, St. Louis, MO, USA). The cells were cultured for 24 h in the presence of the tested compounds (stock solutions of **Tb** and compounds **4**, **5**, **6** were prepared 100 mM in DMSO). Untreated cells were incubated only with the vehicle (DMSO). For comparative purpose of immunomodulatory activity, 100 $pg{\cdot}mL^{-1}$ lipopolysaccharide (LPS) was used. Peritoneal macrophages were maintained at 37 °C, 5% CO_2 in humidified Heraeus incubator. All experimental variants were run in duplicates. The concentration of nitrites was detected in individual, cell-free samples (50 μL) incubated 10 min at ambient temperature with an aliquot of a Griess reagent (1% sulphanilamide/0.1% naphtylendiamine/2.5% H_3PO_4). The absorbance was recorded at 540 nm using a microplate spectrophotometer (Tecan, Austria). A nitrite calibration curve was used to convert absorbance to concentration (μM) of nitrite.

4.4. Statistical analysis

Analysis of variance (ANOVA) and graphical presentation of data were done using GraphPad Prism 6, San Diego, CA.

5. Analytical supplement

5.1. Graphical assignment of the ^{1}H and ^{13}C NMR spectra of the key compounds

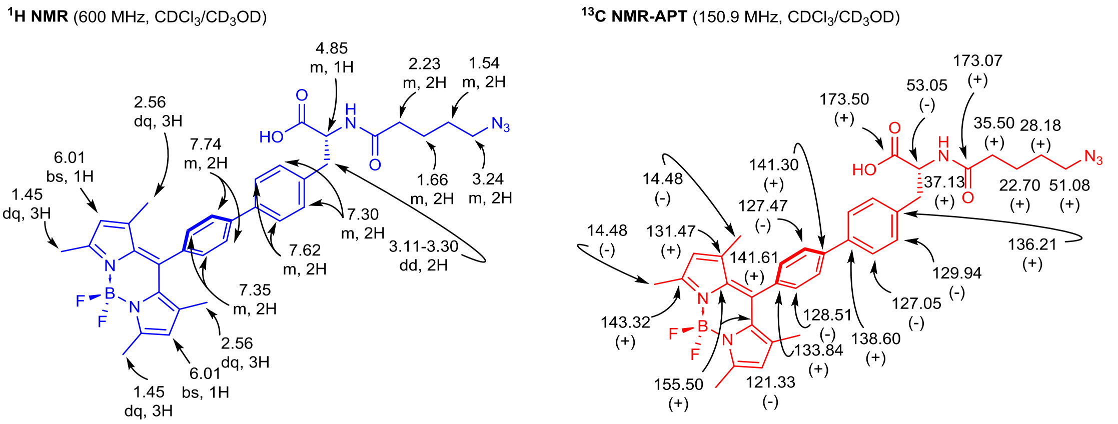

Figure S1. Interpretation of the NMR spectra of compound **3**.

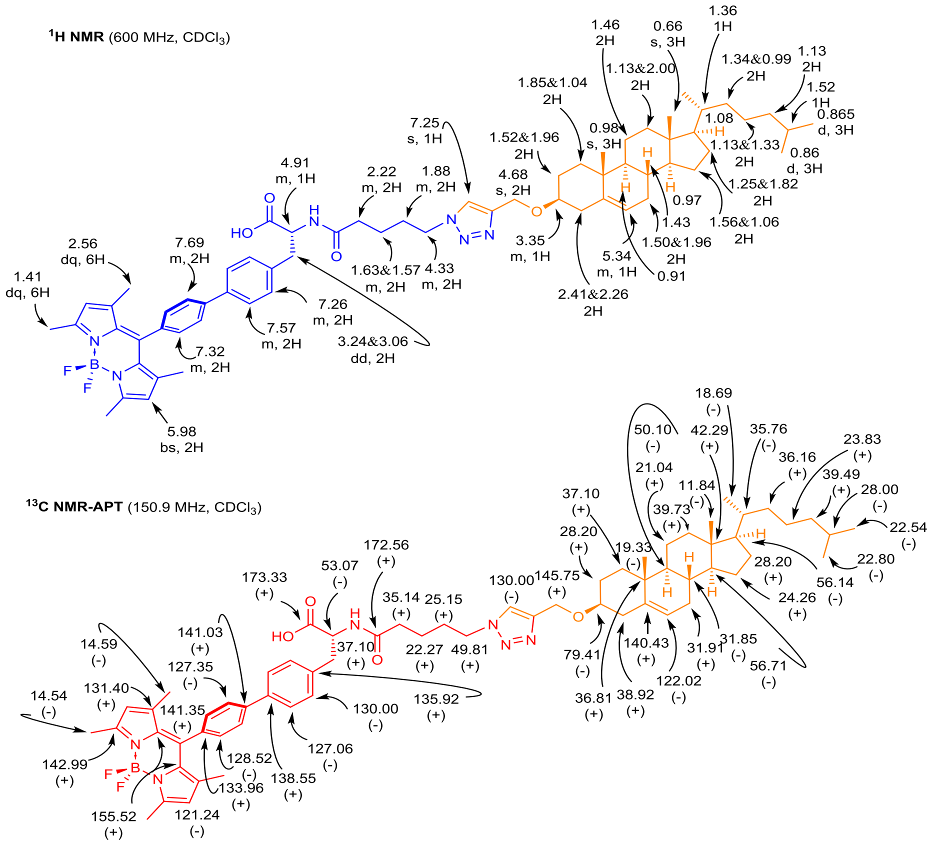

Figure S2. Interpretation of the NMR spectra of compound **4**.

^{1}H NMR (600 MHz, $CDCl_3$)

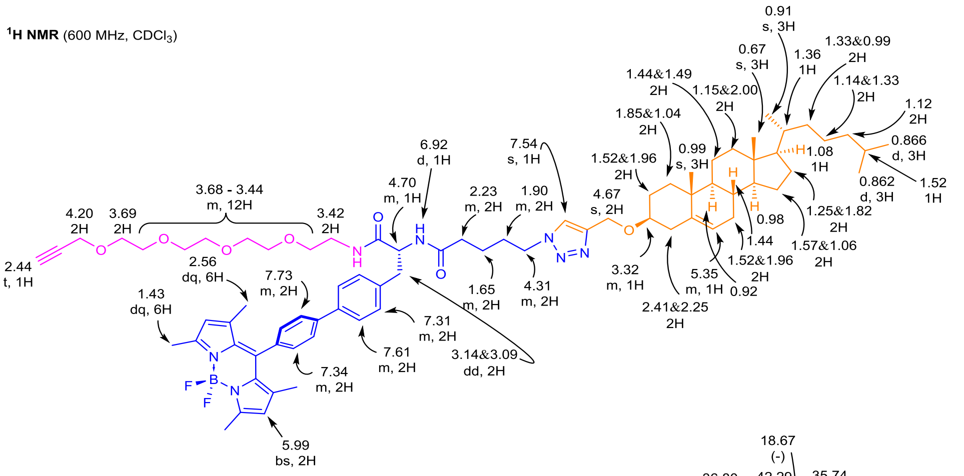

^{13}C NMR-APT (150.9 MHz, $CDCl_3$)

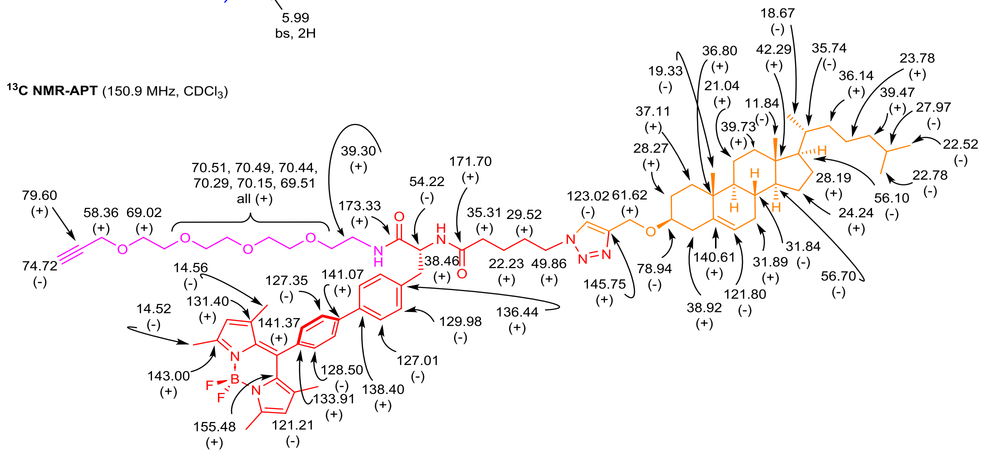

Figure S3. Interpretation of the NMR spectra of compound **5**.

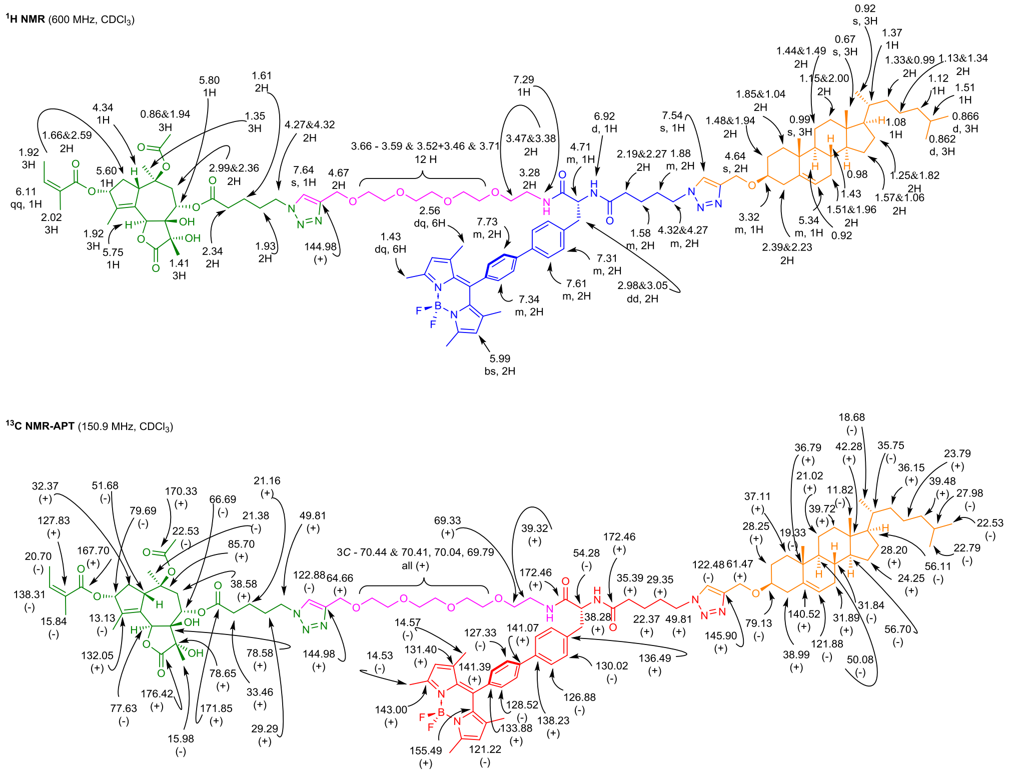

Figure S4. Interpretation of the NMR spectra of construct **6**.

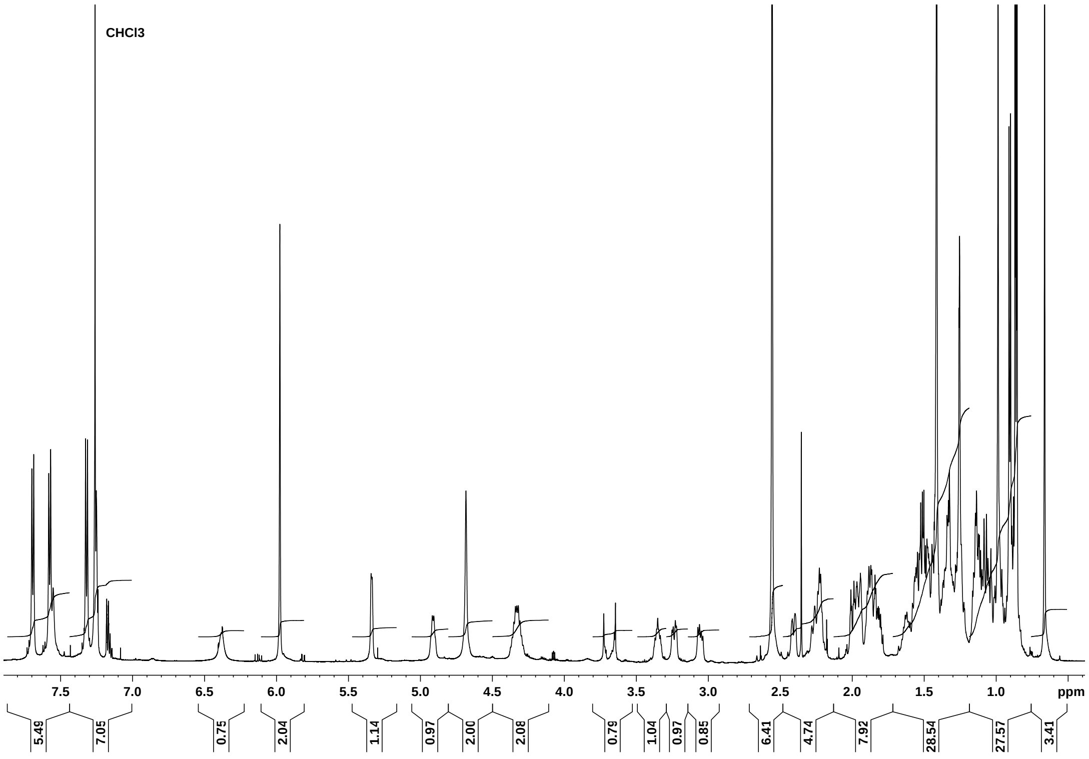

Figure S5. ^{1}H NMR spectrum (600 MHz) of compound **4** $CDCl_3$.

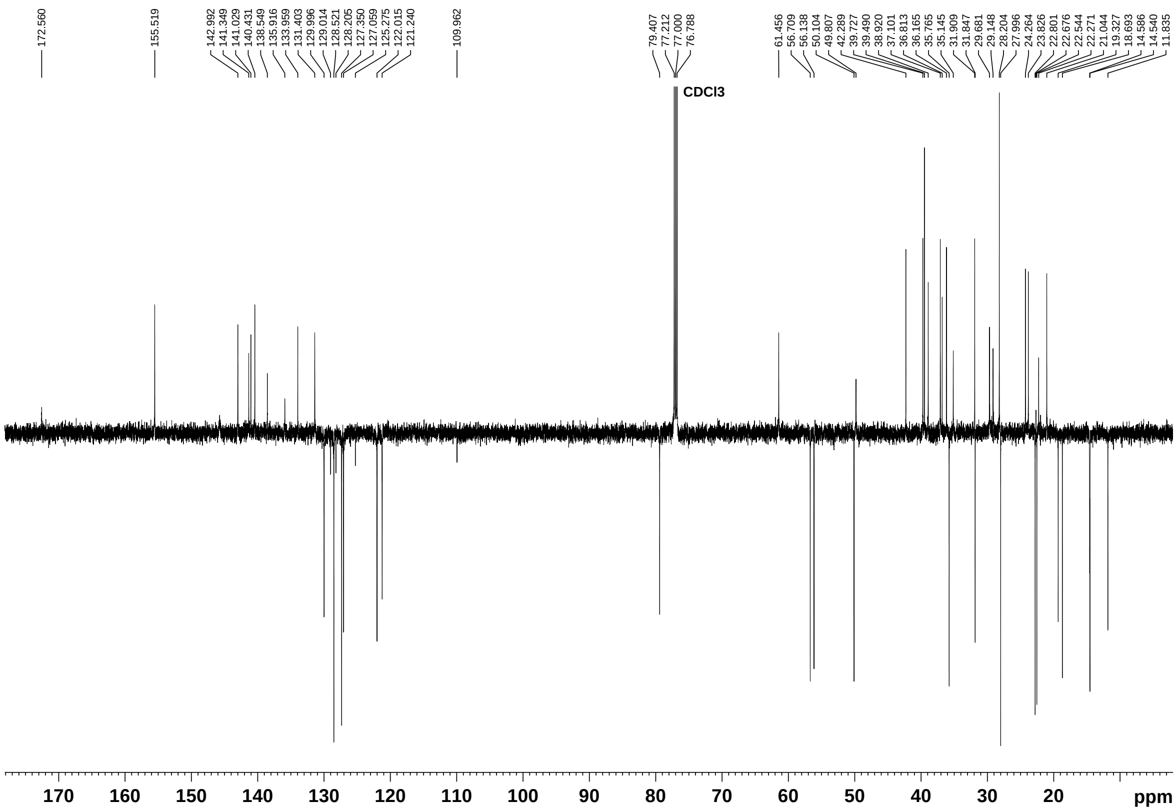

Figure S6. ^{13}C NMR spectrum (150.9 MHz) of compound **4** in $CDCl_3$.

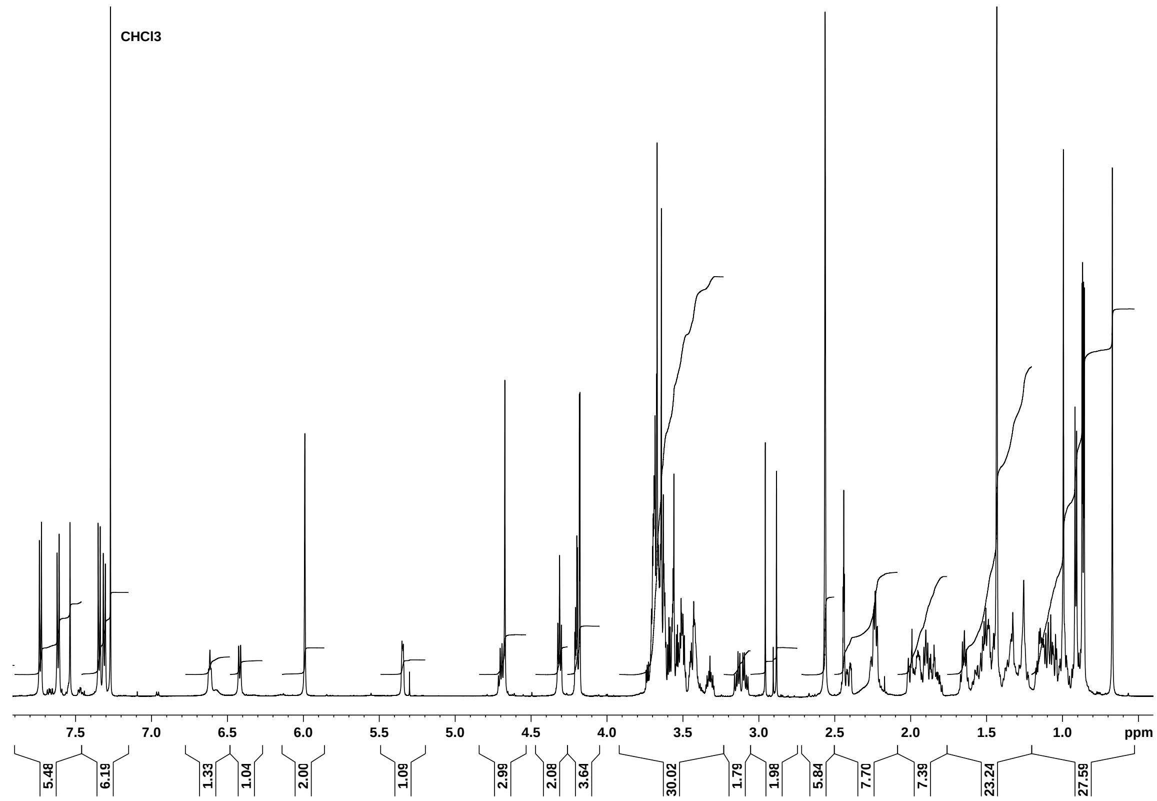

Figure S7. ^{1}H NMR spectrum (600 MHz) of compound **5** in $CDCl_3$.

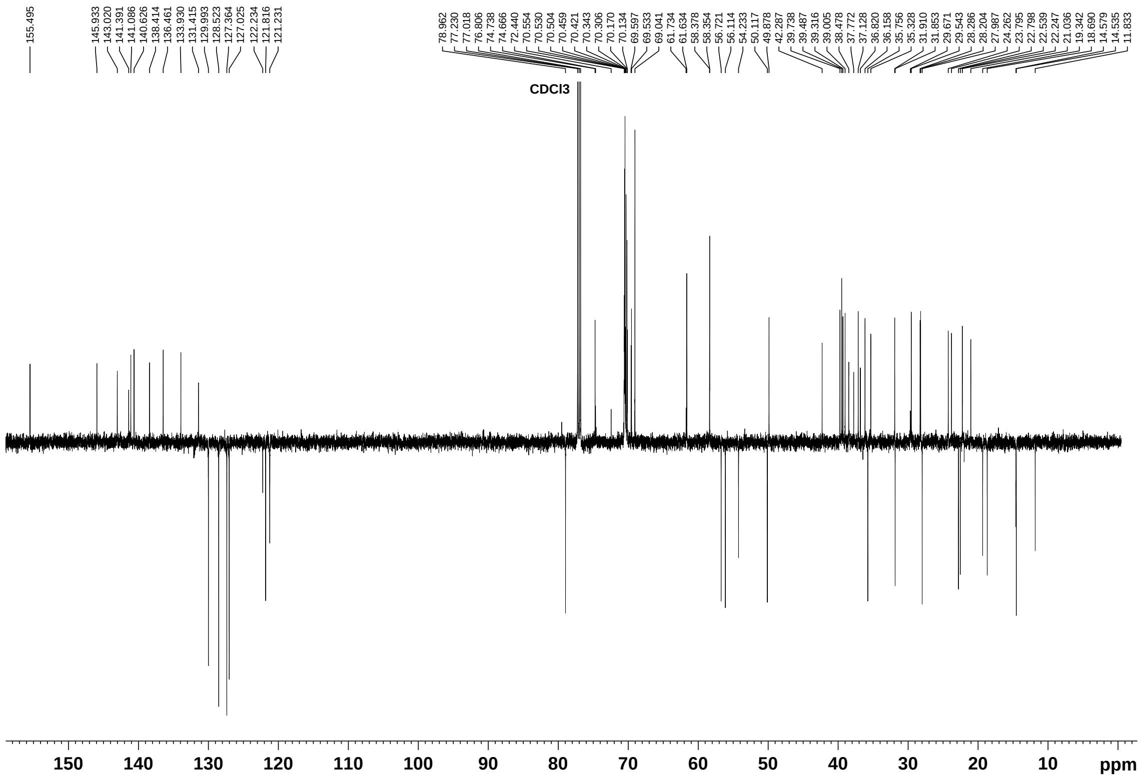

Figure S8. ^{13}C NMR spectrum (150.9 MHz) of compound **5** in $CDCl_3$.

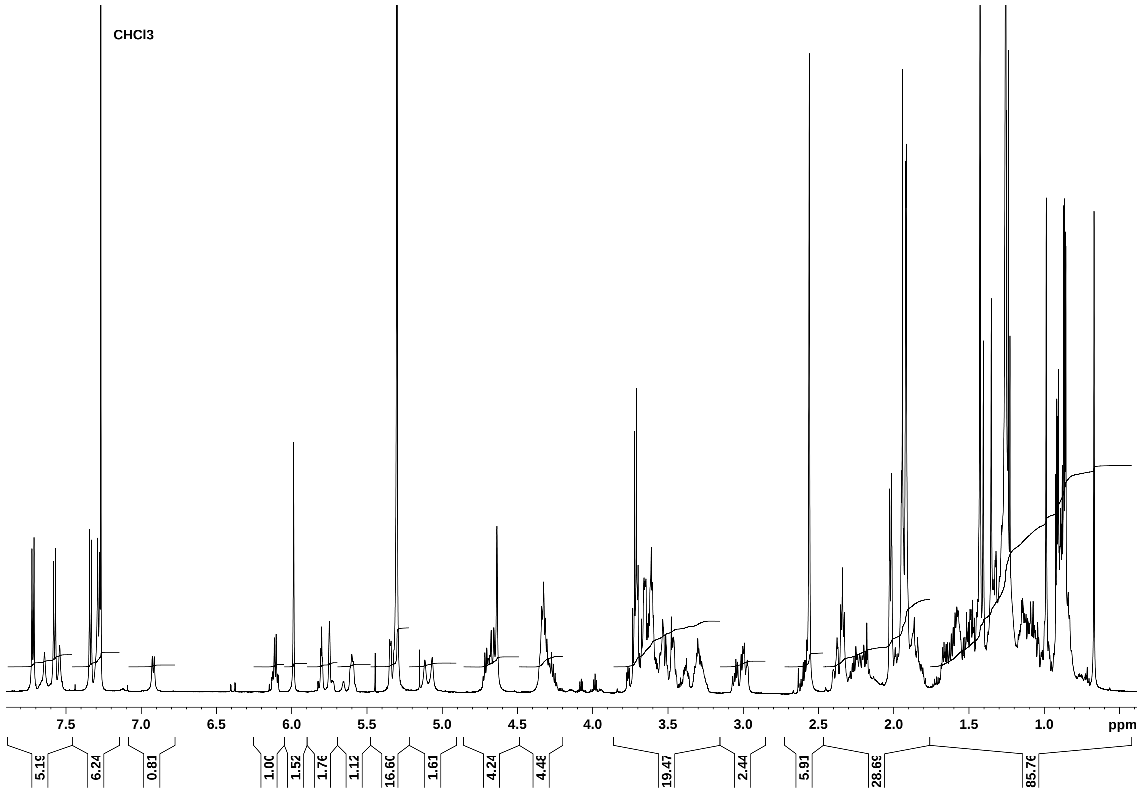

Figure S9. ^{1}H NMR spectrum (600 MHz) of construct **6** in $CDCl_3$.

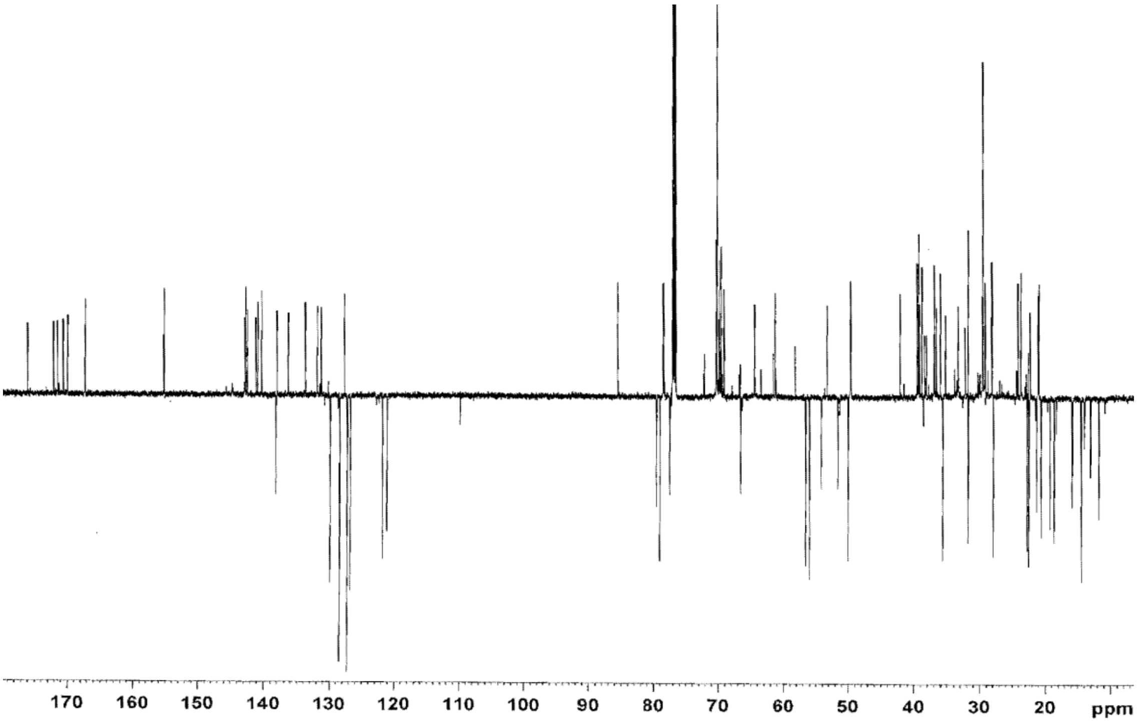

Figure S10. ^{13}C NMR spectrum (150.9 MHz) of construct **6** in $CDCl_3$.

5.2. High-resolution mass spectra

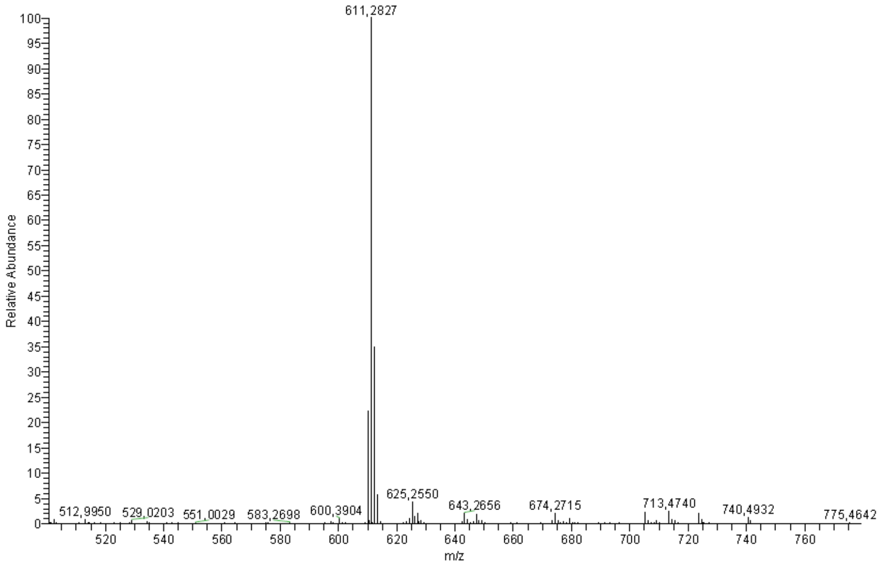

Figure S11. HRMS-ESI spectra of compound **3**.

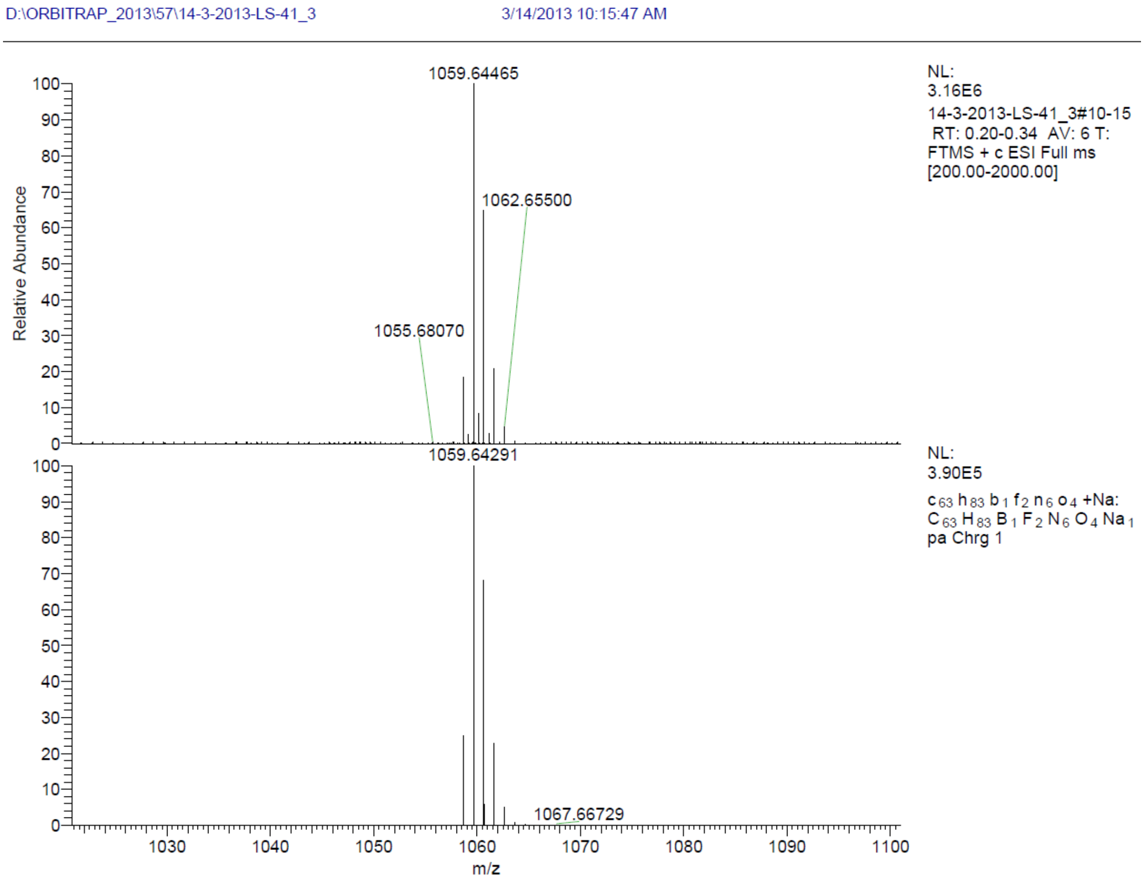

Figure S12. HRMS-ESI spectra of compound **4**.

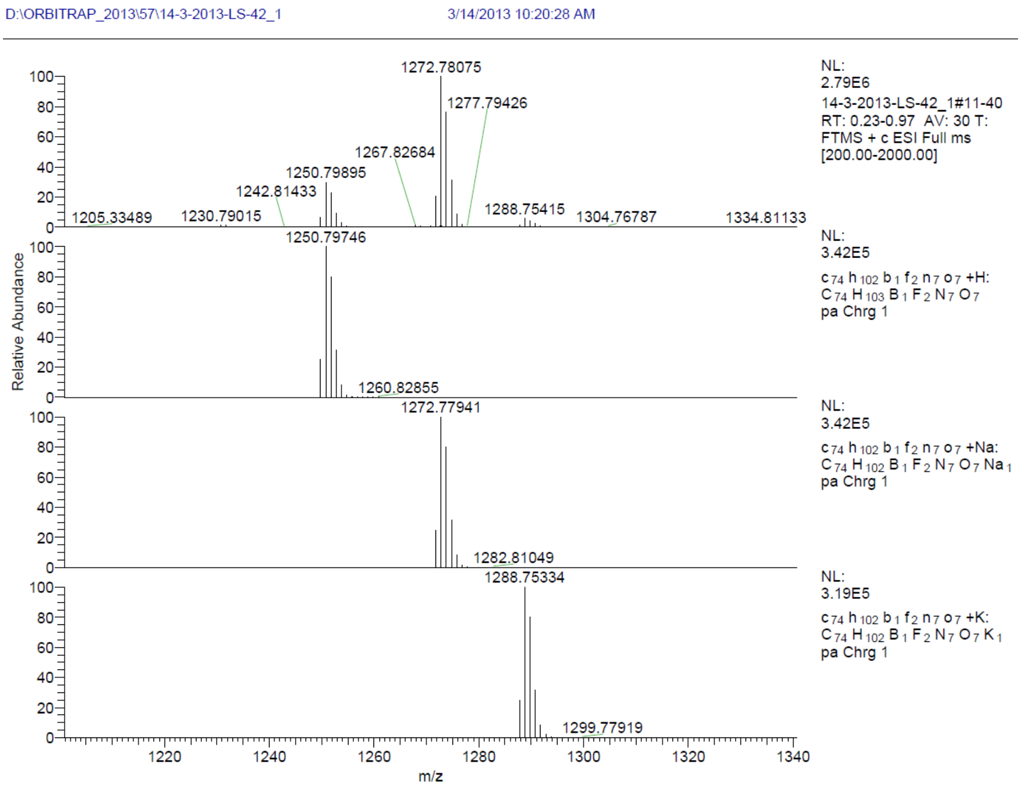

Figure S13. HRMS-ESI spectra of compound **5**.

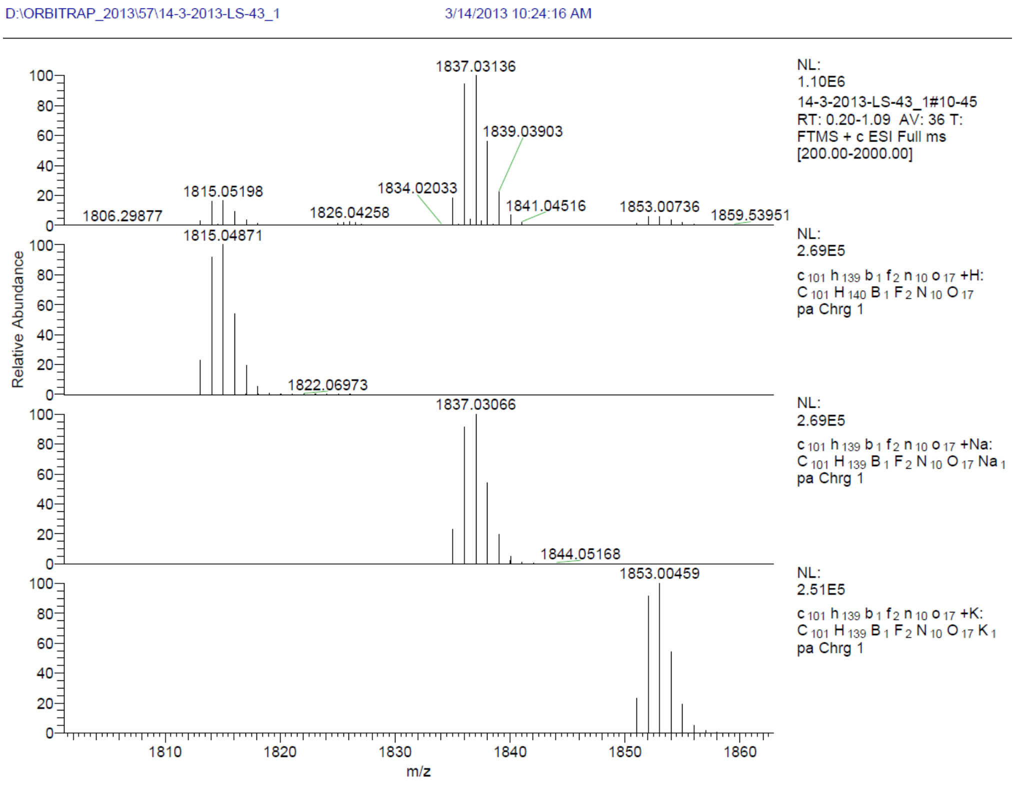

Figure S14. HRMS-ESI spectra of construct **6**

5.3. HPLC chromatogram of the target construct 6

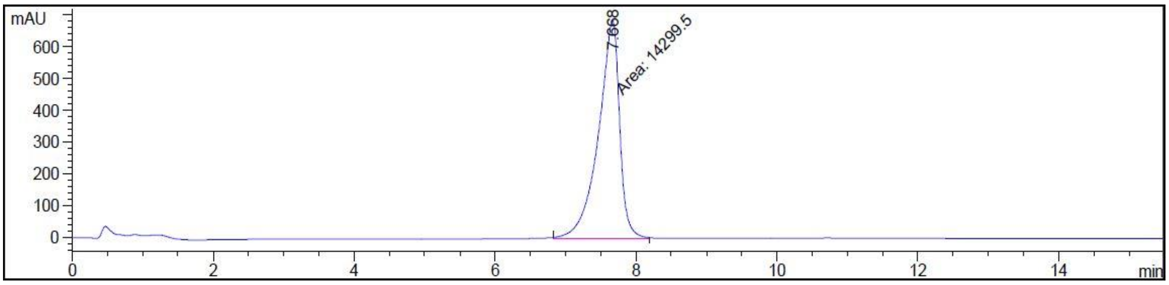

Figure S15. HPLC-MS analysis of the final construct **6** (column RP C-18 Cromasil, flow 0.4 mL/min, 50% MeOH / MeOH gradient (time (min) MeOH (%)); 1/50; 2/60; 3/70; 4/90; 5/100; 15/100).

6. Live-cell imaging

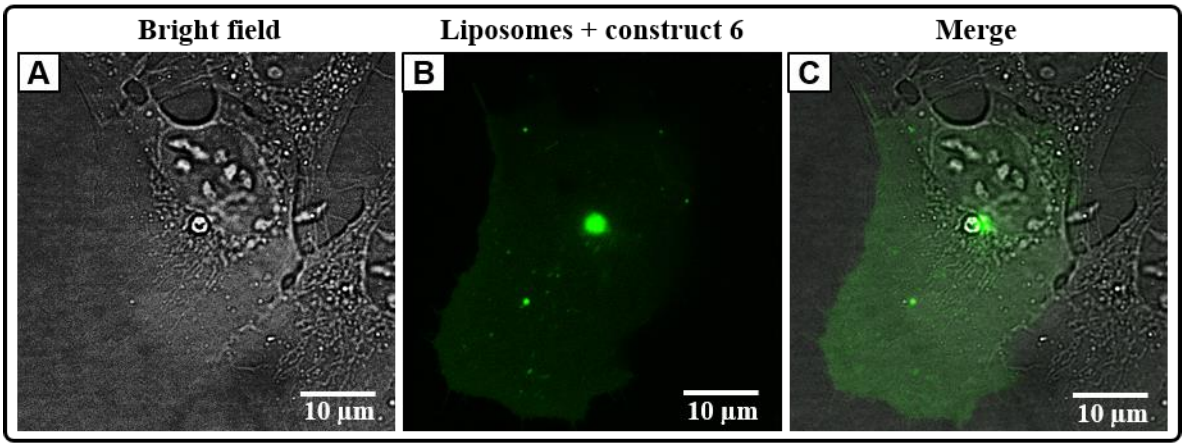

Figure S16. A panel of images from live-cell fluorescence microscopy: intracellular localization of liposomes with construct **6** (43 nM) in U-2 OS cells after 1 h of incubation: A) bright field; B) construct **6** in liposomes; C) merge of images A and B.

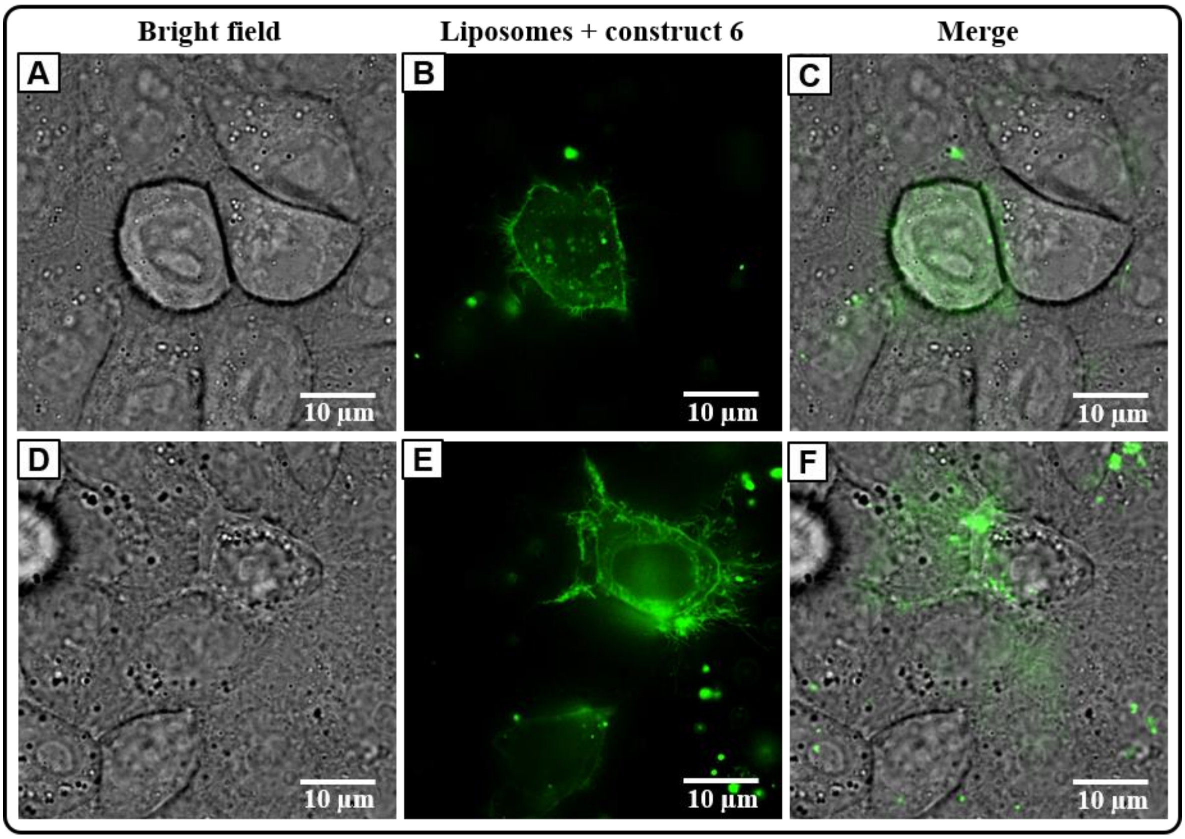

Figure S17. A panel of images from live-cell fluorescence microscopy: intracellular localization of liposomes with construct **6** (125 nM) in HeLa cells after 2 h of incubation: A, D) bright field; B, E) construct **6** in liposomes; C, F) merges of images A and B and D and E.

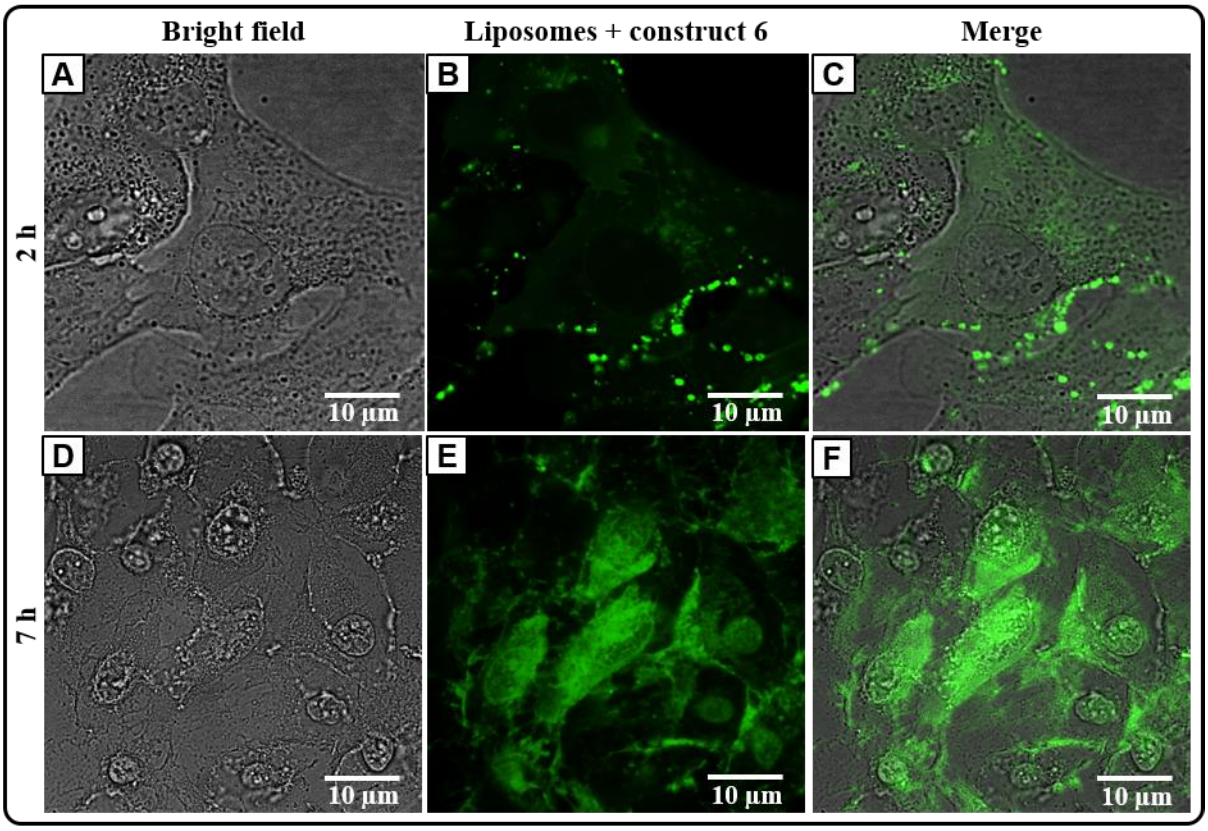

Figure S18. A panel of images from live-cell fluorescence microscopy: intracellular localization of liposomes with construct **6** (1.25 µM) in U-2 OS cells after 2 and 7 h of incubation: A, D) bright field; B, E) construct **6** in liposomes; C, F) merges of images A and D and B and E.

References

[1] Rega, M.; Jimenez, C.; Rodriguez, J. *Steroids* **2007**, *72*, 729-735.

[2] Jurášek, M.; Rimpelová, S.; Kmoníčková, E.; Drašar, P.; Ruml, T. *J. Med. Chem.* **2014**, *57*, 7947-7954.

[3] Bai, M.; Huang, J.; Zheng, X.; Song, Z.; Tang, M.; Mao, W.; Yuan, L.; Wu, J.; Weng, X.; Zhou, X. *J. Am. Chem. Soc.* **2010**, *132*, 15321-15327.
